# Portable Atmospheric Air Plasma Jet Pen for the Surface Treatment of Three-Dimensionally (3D)-Printed Electrodes

**DOI:** 10.1021/acs.analchem.4c02785

**Published:** 2024-09-05

**Authors:** Gilvana P. Siqueira, Raquel G. Rocha, Amanda B. Nascimento, Eduardo M. Richter, Rodrigo A. A. Muñoz

**Affiliations:** Chemistry Institute, Federal University of Uberlândia, 38400-902 Uberlândia, Minas Gerais, Brazil

## Abstract

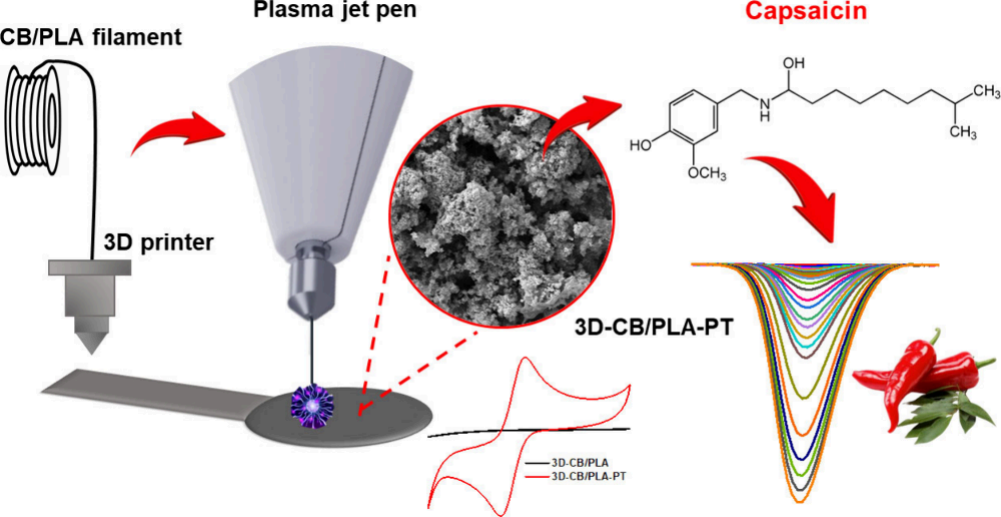

Three-dimensional (3D) printing is an emerging technology to develop devices on a large scale with potential application for electroanalysis. However, 3D-printed electrodes, in their native form, provide poor electrochemical response due to the presence of a high percentage of thermoplastic polymer in the conductive filaments. Therefore, surface treatments are usually required to remove the nonconductive material from the 3D-printed electrode surfaces, providing a dramatic improvement in the electroanalytical performance. However, these procedures are time-consuming, require bulky equipment, or even involve non-eco-friendly protocols. Herein, we demonstrated that portable and low-cost atmospheric air plasma jet pens can be used to activate electrodes additively manufactured using a commercial poly(lactic acid) filament containing carbon black as conductive filler, improving the electrochemical activity. Remarkable electrochemical results were obtained (voltammetric profile) using [Fe(CN)_6_]^3–/4–^, dopamine and [Ru(NH_3_)_6_]^2+/3+^ as redox probes. Microscopic, spectroscopic, and electrochemical techniques revealed that the air-plasma jet pen removes the excess PLA on the 3D-printed electrode surface, exposing the conductive carbon black particles and increasing the surface area. The performance of the treated electrode was evaluated by the quantification of capsaicin in pepper sauce samples, with a limit of detection of 3 nM, suitable for analysis of food samples. Recovery values from 94% to 101% were obtained for the analysis of spiked samples. The new treatment generated by a plasma jet pen is an alternative approach to improve the electrochemical activity of 3D-printed electrodes that present sluggish kinetics with great advantages over previous protocols, including low-cost, short time of treatment (2 min), environmentally friendly protocol (reagentless), and portability (hand-held pen).

## Introduction

Fused deposition modeling (FDM) is a process used to mass-scale production of tailor-made and cost-effective three-dimensional (3D) objects.^1−4^ Several areas including electrochemistry have been benefited by the 3D printing technology.^5^ In this field, FDM has been used to construct batteries, electrochemical cells, sensors, and capacitors.^1,6−8^

Developing electrochemical sensors requires conductive filaments made from a mix of conductive fillers and polymers.^9,10^ Conductive commercial filaments based on a mix of materials, such as graphene/polylactic acid (PLA) (Black Magic) and carbon black/PLA (Protopasta), have been explored in the development of electrochemical sensors. Nevertheless, these filaments contain a high percentage of nonconductive thermoplastic material (∼80%–90%) to ensure proper printability with FDM 3D printers. As a result, 3D-printed electrodes often show poor electrochemical response when “as printed”, with ill-defined voltammetric profiles and low peak current intensities for redox probes, compared with carbonaceous materials such as glassy carbon, carbon nanotubes, and carbon paste.^9,11,12^

Several strategies have been explored to reduce the insulating polymer on 3D-printed electrode surfaces. These methods enhance conductive agent exposure and increase porosity, improving the electron transfer kinetics and the performance of the electrochemical sensors.^9,11,13−16^ Rocha and colleagues showed a detailed review about the different strategies for treatment or activation of 3D-printed electrode surfaces.^5^ Although these procedures improve the electrochemical response of 3D-printed sensors, most of them employ multistep procedures, toxic organic solvents, and bulky, nonportable, and costly laser and plasma equipment.^12,13,17^ In this sense, an environmentally friendly (reagentless), fast, and reproducible surface treatment is demanded for this purpose.

Hand-held atmospheric air plasma jet pens are commercially available to treat aesthetic affections to the skin.^18^ The application of plasma through pens using electric arc production to ionize gases contained in the atmosphere has made this technology easily accessible.^19^ Using the “cold plasma” (room-temperature pen), these devices excite the gases around them through an energy source capable of producing a high-voltage or high-frequency electric field, which is applied between the tip (i.e., cathode) and the surface to be treated (anode). The air dielectric barrier (insulator) causes an electrical discharge, usually in the form of sparks or arcs, and thus generates a luminous plasma associated with the pen.^18^ The plasma generated by plasma jet pens is very similar to that described in the literature with the name “dielectric barrier discharge (DBD) plasma”, which is characterized by enabling the generation of plasma at room temperature and normal atmospheric pressure without the need for a vacuum. DBD plasma stands out for being a relatively simple and economical technique.^18,20^ As far as we know, there are no investigations of the effect of atmospheric air plasma generated by “plasma jet pens” on the surface of 3D-printed electrodes and their respective electrochemical activity. Moreover, such an investigation has not been reported for other electrodes either.

Thus, we demonstrate that atmospheric air plasma jet pens provide a substantial improvement of the electrochemical activity of 3D-printed electrodes, resulting in outstanding sensing properties. This treatment is a new, low-cost, fast (2 min), and environmentally friendly/portable approach (battery powered and use of atmospheric air) to remove excess PLA from the surface of electrodes. As a proof-of-concept, the 3D-printed treated electrodes were applied for the electrochemical determination of capsainoid profile in pepper sauces.^21^ Capsaicin and dihydrocapsaicin are the two most active components of capsaicinoids (∼90%). The total capsaicinoid content is a key quality control parameter for peppers, directly correlating with their heat (pungency) level.^22−26^

## Experimental Section

The complete experimental section is described in more detail in Section 1, presented in the Supporting Information.

### Treatment of Carbon Black and PLA Electrodes Using an Atmospheric Air Plasma Jet Pen

A commercial carbon black/PLA (CB/PLA) filament was used to fabricate 3D-printed electrodes (printing conditions in Table S1), which were then treated with an atmospheric air plasma jet pen (PLASMAX-EHF 2204, KLD Biosistemas, São Paulo, Brazil). Electrode dimensions are listed in Figure S3. For surface activation of the 3D-CB/PLA electrode by the plasma jet pen, the conditions for plasma generation were controlled, considering the plasma generation in continuous mode and the distance between the tip of the plasma jet pen and the surface of the 3D-CB/PLA electrode was <1 mm. Air plasma jet pens (see the scheme in Figure S1) present some parameters that influence the plasma generation, which consequently affect the electrochemistry of the treated electrodes. In this way, the surface treatment of 3D-printed electrodes (illustrated in Figure S2) was systematically investigated by using a plasma jet pen. Details on parameter selection are presented in the Supporting Information (Section 2, Figure S4 and Tables S2 and S3). The following optimized treatment conditions with the generated plasma were used to activate the 3D-CB/PLA electrode surface: application mode (horizontal lines), application time (2 min), plasma power (3000 mW), and type of needles (needle holder, corresponding to a holder with a 0.20 × 15 mm needle fitting). The surface treatment of a 3D-printed electrode using a plasma jet pen is shown in the following YouTube video: https://youtu.be/-LoNBHtiBRU.

## Results and Discussion

### Surface Treatment of 3D-Printed Electrodes Using Plasma Jet Pen

**Figure 1:**
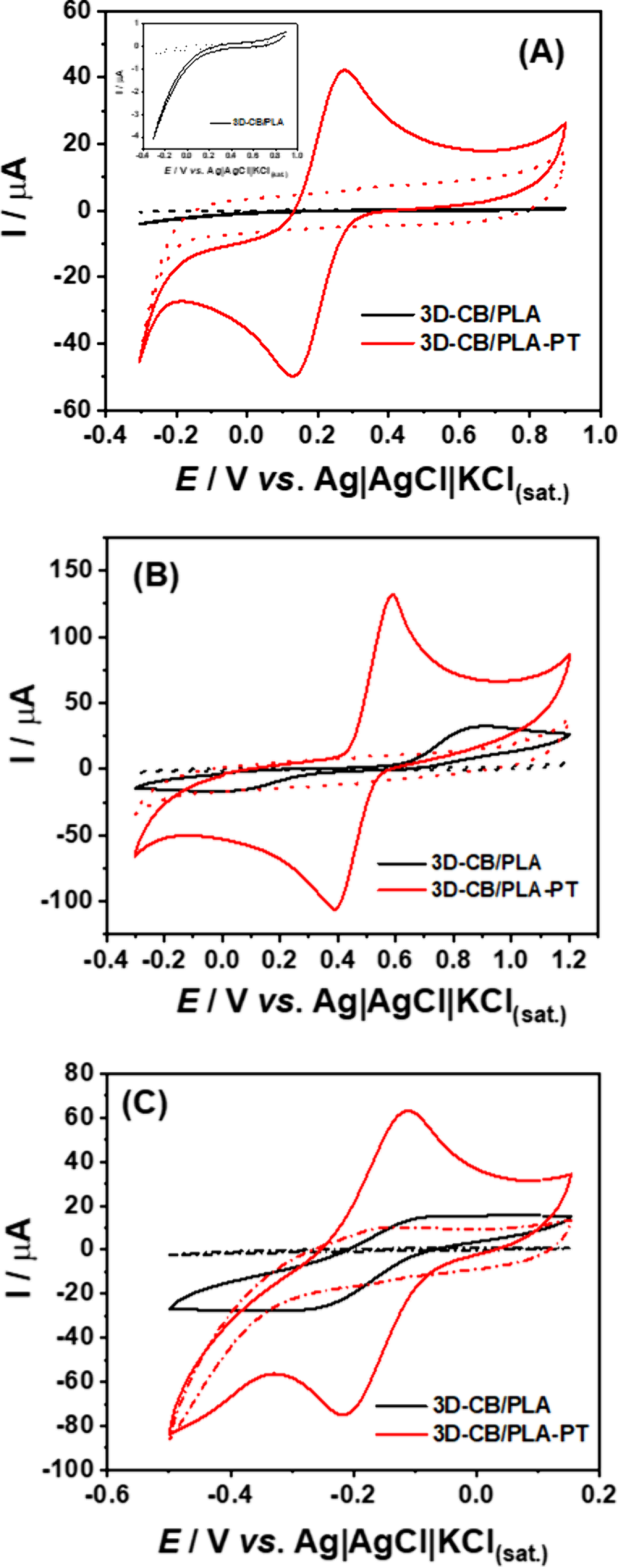
CV data obtained at 3D-CB/PLA (black line) and 3D-CB/PLA–PT (red line) electrodes for (A) 2 mM [Fe(CN)_6_]^3–/4–^ in 0.1 M KCl, (B) 1 mM DOP in 0.1 M HClO_4_, and (C) 2 mM [Ru(NH_3_)_6_]^2+/3+^ in 0.1 M KCl. The dashed lines correspond to the respective blank signals. CV conditions: scan rate = 50 mV s^–1^; step potential = 5 mV.

Figure 1A shows the cyclic voltammograms (CVs) of 2 mM [Fe(CN)_6_]^3–/4–^ using non-treated (3D-CB/PLA) and air-plasma jet-pen treated (3D-CB/PLA–PT) electrodes under optimum conditions. Figure S3 shows real images of both non-treated and treated electrodes. As can be seen, the 3D-CB/PLA electrode displayed a poor voltammetric profile (characteristic redox peaks are not visible) for this probe. This behavior was expected as previously stated for inner-sphere redox probes, such as the case of [Fe(CN)_6_]^3–/4–^, and originates from the low conductivity of the CB/PLA filament.^27^ However, when the 3D-printed electrode was subjected to the air-plasma jet pen treatment, a better voltammetric profile was obtained with a higher current response and lower peak-to-peak separation (Δ*E*_p_), typically observed in conventional carbon electrodes.

The reproducibility of different 3D-CB/PLA–PT electrodes was assessed with CV measurements in the presence of 2 mM [Fe(CN)_6_]^3–/4–^ (*n* = 4; see Figure S5). Table S4 shows their respective Δ*E*_p_ and *I*_pa_/*I*_pc_ values for each 3D-CB/PLA–PT electrode. The relative standard deviation (RSD) values were 10.6% and 1.6% for Δ*E*_p_ and *I*_pa_/*I*_pc_, respectively. Importantly, the average values of Δ*E*_p_ (144 mV) and peak current ratio (*I*_pa_/*I*_pc_ = 1.09) are enhanced, in comparison with other surface treatment protocols applied for 3D-printed electrodes, considering the same redox probe and the same commercial conductive filament. Table S5 summarizes this comparison to the literature. As noticed, some activation protocols involve organic solvent or costly reagents, are time-consuming, or use bulky and costly equipment. Although CO_2_ laser-scribing treatment resulted in lower Δ*E*_p_ (130 mV for [Fe(CN)_6_]^3–/4–^) than the proposed air-plasma treatment, the CO_2_ cutter equipment for laser-scribing is more expensive, bulky, and not portable.^28^

The dopamine (DOP) electrochemical response was checked (Figure 1B, and Table S6). As expected, an ill-defined voltammetric profile (Δ*E*_p_ = 814 mV and *I*_pa_/*I*_pc_ = 2.12) was observed using nontreated 3D-printed CB/PLA electrodes. However, the DOP response significantly improved after electrode surface treatment, leading to well-defined and sharp peaks and lower Δ*E*_p_ (Δ*E*_p_ = 191 mV and *I*_pa_/*I*_pc_ = 1.09). The Δ*E*_p_ was reduced 400% when compared to non-treated electrodes. Comparing this result with other surface treatment protocols reported for DOP detection, Pereira et al. reported a Δ*E*_p_ of 283 mV for a CO_2_ plasma-treated carbon black PLA electrode^17^ and Cardoso et al. found a Δ*E*_p_ at ∼300 mV using a 3D-printed graphene PLA electrode treated by mechanical polishing.^29^ Crapnell and co-workers^30^ demonstrated the determination of DOP using a lab-made filament based on recycled PLA, carbon black, and castor oil as a plasticizer. Before use, the 3D-printed electrodes were subjected to an electrochemical treatment procedure in alkaline medium and a Δ*E*_p_ value of ∼200 mV was observed. Hence, it is worth mentioning that the voltammetric performance of the proposed 3D-CB/PLA–PT for DOP is very similar or better than previously reported 3D-printed electrodes that have been subjected to different surface treatment protocols.

Furthermore, CV results using an outersphere [Ru(NH_3_)_6_]^2+/3+^ probe (Figure 1C) show that the 3D-CB/PLA–PT electrode has a higher current response (∼5-fold) and better reversibility (*I*_pa_/*I*_pc_ = 1.19, Δ*E*p = 91 mV) compared to the 3D-CB/PLA electrode (*I*_pa_/*I*_pc_ = 0.72, Δ*E*_p_ = 156 mV). This suggests that the treatment not only removes PLA but also improves the electrical conductivity of graphitic carbon.

The shelf life of the treated sensor was assessed through daily stability tests, estimating the CV profile of 2 mM [Fe(CN)_6_]^3–/4–^ (Figure S6). The proposed modification maintained the sensor’s lifespan, sustaining consistent electrochemical performance even after 15 days of continuous use, with an RSD of 3.88% for *I*_pa_/*I*_pc_ and 2.86% for Δ*E*p (*I*_pa_/*I*_pc_ = 1.03 ± 0.04, Δ*E*_p_ = 140 ± 4 mV). We also investigated whether polishing in the 3D-CB/PLA–PT electrode would remove the proposed modification. In Figure S7, we demonstrated that the plasma treatment indeed involves superficial modification of the surface. Simple abrasive polishing with water sandpaper can remove this activated surface layer, thereby reducing or nullifying the benefits obtained from the activation process. The results suggest that the electrode can be reused, which is crucial for waste reduction.

Additionally, the performance of the 3D-CB/PLA–PT electrode was compared with electrochemical activation in 0.5 M NaOH solution (3D-CB/PLA-QET electrode) proposed by Richter et al. in 2019,^31^ in 2 mM [Fe(CN)_6_]^3–/4–^ (Figure S8). The 3D-CB/PLA–PT electrode exhibited lower Δ*E*_p_ value of 136 mV and an *I*_pa_/*I*_pc_ value of 1.04, compared to the 3D-CB/PLA-QET electrode (Δ*E*_p_ = 236 mV, *I*_pa_/*I*_pc_ = 0.98). Furthermore, there is an increase of 4.5 times in *I*_pa_ and *I*_pc_, when using the 3D-CB/PLA–PT electrode. These values demonstrate the superior performance of the proposed surface modification.

### Electrode Surface Characterization

**Figure 2:**
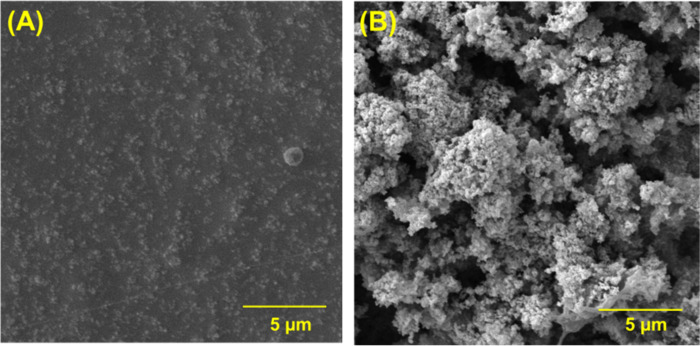
SEM images of (A) 3D-CB/PLA and (B) 3D-CB/PLA–PT electrodes.

Both electrode surfaces were characterized by scanning electron microscopy (SEM) (Figure 2), and different magnifications are shown in Figure S7. The 3D-CB/PLA electrode (Figure 2A and S9A) exhibited a smooth surface, due to the large amount of polymer (∼80% of the filament is composed of PLA) on the surface and CB particles are covered by the polymeric matrix (low availability of conductive particles).^27,32^ After plasma treatment (see Figures 2B and S9B), the porosity of the electrode surface increased considerably with a visible enhancement in the surface area. Also, the existence of sponge-like structures was revealed due to the partial removal of the insulating material (PLA) and the exposure of CB particles by the action of atmospheric air plasma. In addition, the formation of cracks and, consequently, a rough surface, is notable.^11,17^

The atomic force microscopy (AFM) technique was also employed to evaluate the surface treatment effect. Figure S10 presents topographic images of the 3D-CB/PLA surface (Figure S10A) and the 3D-CB/PLA–PT surface (Figure S10B). Before the treatment, a smoother surface is observed, as expected, although it is possible to see lines on the surface caused by the 3D printing process. After plasma treatment, a considerable increase (by a factor of 6) in surface roughness (rms = 54.8) was achieved when compared to untreated electrode (rms = 8.7). These results agreed with the SEM images in which a porous-like morphology with a visible increase in the surface area was observed. Moreover, the increase in the electrochemical response for redox probes (DOP, [Ru(NH_3_)_6_]^2+/3+^ and [Fe(CN)_6_]^3–/4–^) can be associated with the improvement of crack formation (groove surfaces).

**Figure 3:**
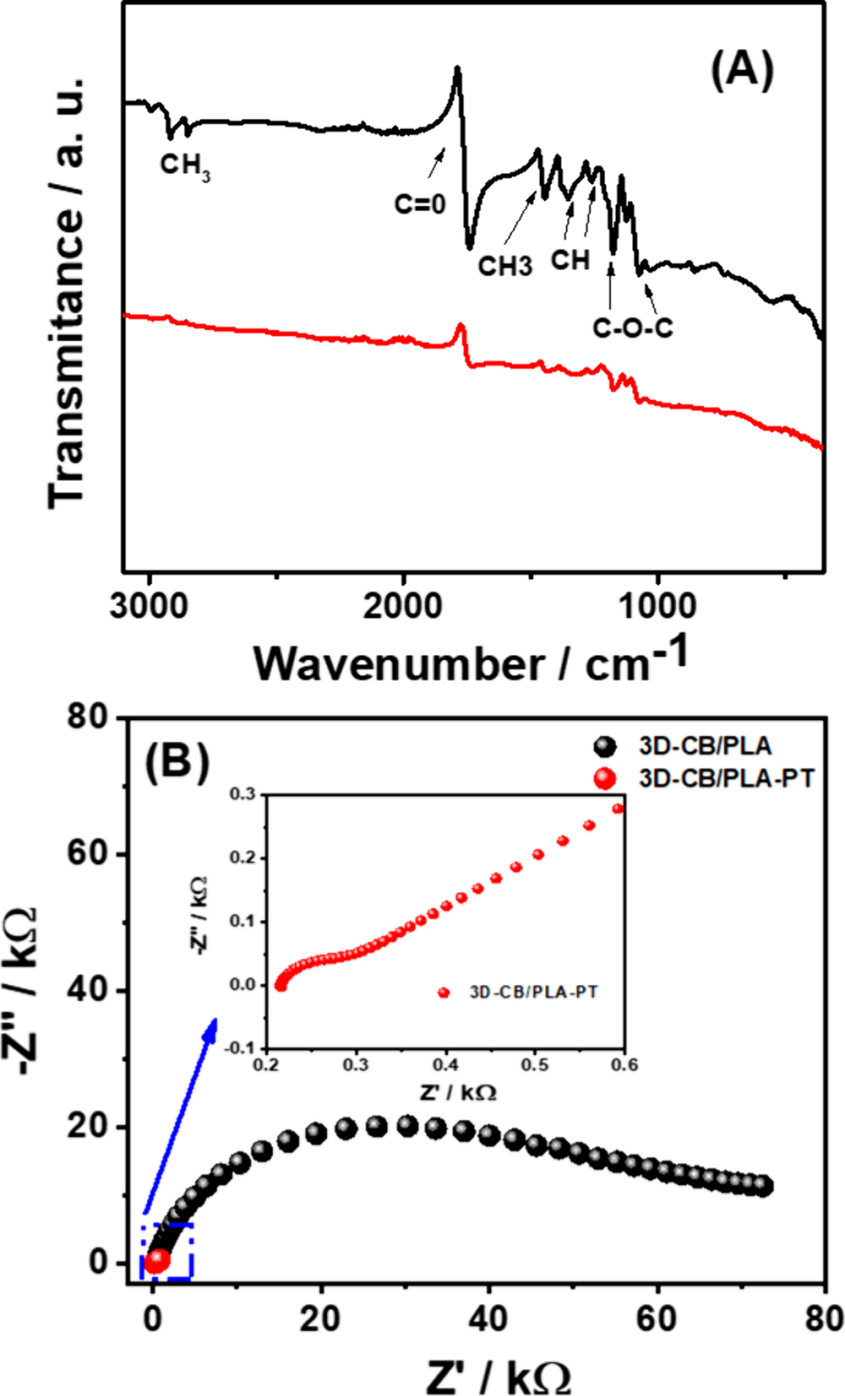
(A) FTIR spectra of the 3D-CB/PLA (black line) and 3D-CB/PLA–PT (red line); (B) Nyquist diagram of impedance spectra at +0.22 V of the 3D-CB/PLA (black dots) and 3D-CB/PLA–PT (red dots) electrodes in the presence of 2 mM [Fe(CN)_6_]^3–/4–^ in 0.1 M KCl solution.

Fourier-transform infrared (FTIR) analyses were also obtained for 3D-CB/PLA (black line) and 3D-CB/PLA–PT (red line) electrodes (Figure 3A). The spectrum of the 3D-CB/PLA electrode displays the main vibrational modes that correspond to the PLA polymer matrix.^17,33^ The low intensity bands at ∼2912 and 1437 cm^–1^ are clearly associated with the antisymmetric and symmetric stretching vibrations of the CH_3_ group, respectively. At 1731 cm^–1^, the high intensity band is associated with the C=O group.^34,35^ The CH strain appears at 1357 cm^–1^, while the bands at 1175 and 1079 cm^–1^ correspond to vibrational modes related to the C–O and C–O–C groups.^34,35^ The surface spectrum of the 3D-CB/PLA–PT electrode reveals a decrease in the PLA band intensities, demonstrating the effectiveness of the treatment through the partial remotion of the polymer on electrode surface, leaving the CB particles more available, thus improving its electron transfer.

Raman spectra of both electrodes were acquired (Figure S11). The presence of D-band (1350 cm^–1^), G-band (1603 cm^–1^), and 2D-band (2820 cm^–1^) peak positions are associated with the presence of carbon black. The D-band is related to the defects and the formation of sp^3^ bonds and oxygenated species. On the other hand, the G-band is associated with C=C stretch in the sp^2^ species.^17,36,37^ The intensity ratio of the *I*_D_/*I*_G_ bands for each electrode was calculated and used as a parameter for the degree of structural defects on the surface.^17,37,38^ The *I*_D_/*I*_G_ values were 0.98 and 1.08 for 3D-CB/PLA and 3D-CB/PLA–PT, respectively, which indicates an increase in structural defects because of the plasma application.

Since the capacitance of the electrical double layer (*C*_dl_) is directly proportional to the electroactive area,^8,39^ we estimated *C*_dl_ by using CV data of a blank solution, as shown in Figures S12A and S12B. The *C*_dl_ value is significantly higher for 3D-CB/PLA–PT (*C*_dl_ = 368.5 μF cm^–2^) when compared to the values for the 3D-CB/PLA (*C*_dl_ = 1.6 μF cm^–2^) in which an increase by a factor of ∼230 was observed, indicating an increase in the electroactive area of the 3D-CB/PLA–PT electrodes (Figure S12C).^13^ Interestingly, the estimated *C*_dl_ corroborates with morphological characterization since these analyses showed a partial remotion of the PLA and an increase in porosity and greater availability of CB particles.

Using data from the scan rate study in [Ru(NH_3_)_6_]^2+/3+^ probe (Figure S13), the electroactive area of both electrodes surfaces was calculated as 0.048 and 0.34 cm^2^, respectively, showing a 7.08-fold increase due to the modification. The 0.34 cm^2^ value for the 3D-CB/PLA–PT electrode exceeds the geometric area (0.22 cm^2^), because of surface porosity and roughness, which are not taken into account by the Randles–Sevcik equation. Thus, the *C*_dl_ values more accurately represent the increased electroactive area of the sensor.

Characterizations by electrochemical impedance spectroscopy (EIS) were also performed for both electrodes (Figure 3B). The charge-transfer resistance (*R*_ct_) values were 53.6 ± 0.8 kΩ and 104.1 ± 9.5 Ω for 3D-CB/PLA and 3D-CB/PLA–PT, respectively. The highest *R*_ct_ value for the 3D-CB/PLA electrode was related to the large amount of insulating material (PLA) in the filament composition,^17,27,33^ which decreases considerably after treatment with atmospheric air plasma, due to the removal of the polymeric material from the electrode surface, exposing CB nanoparticles, which are highly conductive.^38^ The EIS results agreed with the estimation of *C*_dl_ since a visible increase in porosity and consequent greater availability of CB nanoparticles (increase in the effective area) was observed.

### Analytical Performance

As a proof-of-concept, the 3D-CB/PLA–PT electrode was applied for capsaicinoid (CPS) quantification. Details about the CPS electrochemical behavior on the 3D-CB/PLA–PT electrode surface are discussed in the Supporting Information (Section 13, Figures S14–S17). The CPS electrochemical detection on the 3D-CB/PLA–PT electrode was performed by differential pulse voltammetry (DPV) using optimized parameters (amplitude = 80 mV, step potential = −6 mV, and modulation time = 30 ms). The optimization studies are described in Section 14 in the Supporting Information.

**Figure 4:**
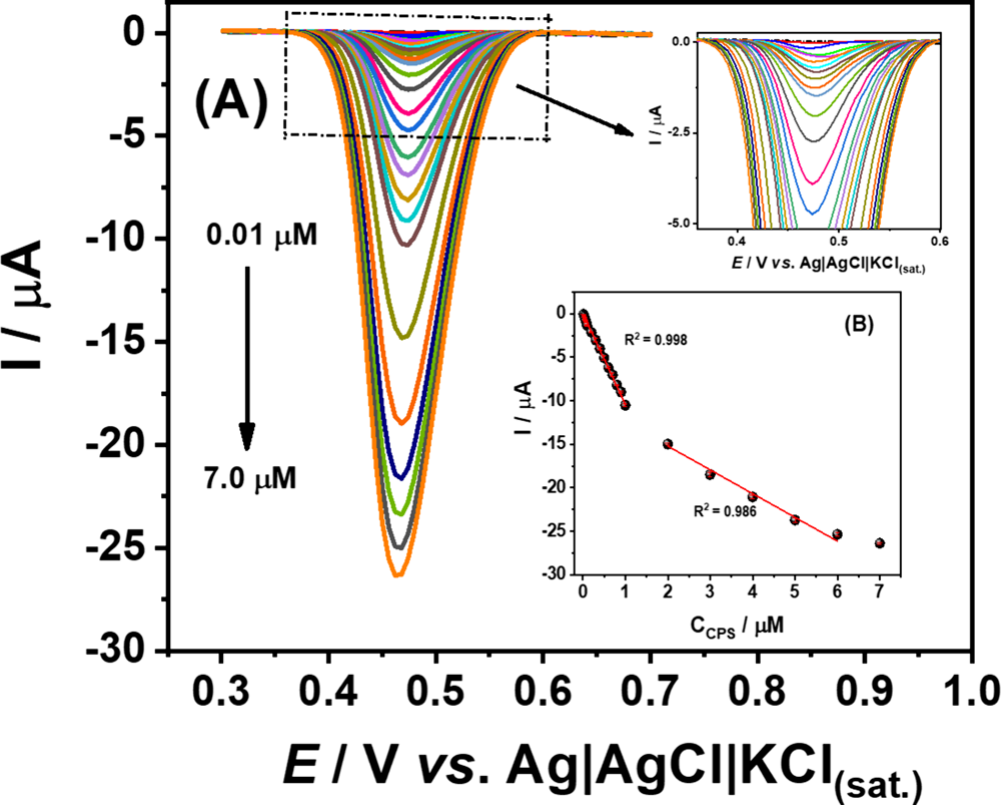
(A) DPV responses obtained for successive increasing concentrations of CPS (0.01–7.0 μM) using 0.12 M Britton–Robinson buffer (pH 2.0) as the supporting electrolyte; (B) Respective calibration plots. DPV conditions: amplitude = 80 mV, step potential = −6 mV, modulation time = 30 ms, and scan rate = 12 mV s^–1^.

Figure 4A shows the DPV responses obtained for increasing concentrations of CPS. A well-defined peak at around +0.5 V is observed for all concentrations. The cathodic peak currents increased linearly with the CPS concentration, and two linear ranges (0.01–1.0 and 2.0–6.0 μM) were observed (see Figure 4B, inserted plot). Two linear ranges for CPS arise from surface saturation of the 3D-CB/PLA–PT electrode at concentrations above 1.0 μM. On rough surfaces, the analyte deposits both inside and on the irregular structure. At lower concentrations, the accessible active surface area becomes fully covered, enhancing sensitivity.^40^ Limit of detection (LOD) and limit of quantification (LOQ) values were estimated as 0.003 and 0.011 μM, respectively, using the first linear range and IUPAC guidelines.^41^ The obtained LOD is appropriate to determine the CPS in real food samples.

The repeatability (Figures S19A and S19B) of the 3D-CB/PLA–PT electrode was assessed by successive DPV measurements (*n* = 10) of two CPS concentration levels (0.05 and 0.1 μM). The RSD values were 2.3% and 1.9%, respectively, which indicates good precision of the method. The reproducibility of the electrochemical proposed method was also verified using four different 3D-CB/PLA–PT electrodes in the presence of 0.1 μM CPS (Figures S19C). RSD values of 3.0% for peak currents (1.89 ± 0.06 μA) and 1.1% for peak potentials (493 ± 6 mV) were obtained (Table S8). These results are considered adequate (RSD ≤ 3%) for analytical applications.^38,42^ The analytical parameters obtained for CPS quantification are listed in Table S9.

The stability of the 3D-CB/PLA–PT sensor was assessed over 100 measurements in the presence of 0.5 μM CPS within a single day (Figure S20). Minimal variation in peak current (RSD = 1.06%) was observed across 101 consecutive measurements. Additionally, the sensor surface exhibited excellent reversibility values for 2 mM [Fe(CN)_6_]^3–/4–^ both before and after the 101 measurements with an RSD of 1.89% for *I*_pa_/*I*_pc_ and 2.86% for Δ*E*_p_ (*I*_pa_/*I*_pc_ = 1.06 ± 0.02, Δ*E*_p_ = 140 ± 4 mV), demonstrating that there was no change in the surface.

Furthermore, when comparing the electrochemical response of the proposed 3D-CB/PLA–PT sensor with the 3D-CB/PLA-QET (see Figure S21) in the presence of 5.0 μM CPS by DPV measurements, an increase (3.6-fold) was observed for the 3D-CB/PLA–PT sensor.

Table S10 compares the performance of the proposed 3D-printed sensor to other electrochemical CPS sensors reported in the literature. The limit of detection (LOD) of the 3D-CB/PLA–PT electrode proposed is superior and/or comparable to most previous reported electrochemical methods. Furthermore, most electrochemical sensors involve laborious and/or costly modification steps. The proposed surface treatment is simple and efficient, and 3D-printed electrodes can be fabricated at low cost ($ 0.20 each sensor) and in a bespoke design. Although the treatment of 3D-CB/PLA was required, the use of plasma jet pen is simple, portable, environmentally friendly (reagentless), and easy to reproduce. Additionally, the pen used for the treatment procedure is low-cost ($100–400) and can be used to perform 30 modifications per hour under optimized conditions. The plasma jet pen features an internal rechargeable lithium-ion battery with a capacity of 2700 mAh, offering up to 4 h of battery life.

To check the accuracy of the proposed method, spike-recovery experiments were performed. For this purpose, CPS was determined in four commercial samples of red pepper sauces (samples A–D) before and after being spiked with CPS. The standard addition method was used to determine the concentration level of CPS in the real samples (Figures S22–S25). The CPS concentrations and recovery values obtained in the analyzed pepper sauce samples are presented in Table S11. Adequate recovery values (ranging from 94% to 101%) for the samples (A_F1_, A_F2_, B_F1_, B_F2_, C_F1_, C_F2_, D_FI_, and D_F2_) demonstrate that the sample matrix did not present significant interference in the proposed method. Therefore, we can infer that CPS can be determined in food products by using the proposed 3D-CB/PLA–PT sensor.

The CPS concentration can be used to determine the total capsaicinoid content in the samples. Commercial products contained 5.07, 3.05, 4.43, and 3.02 mg/L (samples A, B, C, and D, respectively), corresponding to 126.74, 76.35, 110.71, and 75.59 μg of capsaicinoid per gram of pepper sauce. This equates to 0.04–0.06% (w/w), capsaicinoid by weight, which is considered to be mildly hot.^21,43^

The heat or pungency of peppers and hot sauces is typically measured using the Scoville Organoleptic Test,^44^ which is often questioned for its inaccuracy and subjectivity, as it relies on the opinions of tasters,^25^ demonstrating their fragility in monitoring and evaluating food quality and safety.^45,46^ The proposed method using a 3D-CB/PLA–PT sensor stands out for its accuracy when analyzing the capsaicinoid profile of pepper sauces.

## Conclusion

We demonstrate, for the first time, the use of atmospheric air plasma generated via a jet pen to treat and improve the electrochemical performance of 3D printed CB/PLA electrodes. According to the characterizations results (SEM, FTIR, Raman, AFM, EIS, CV, and *C*_dl_ measurements), the innovative proposed treatment promotes the removal of PLA and exposes more CB particles, which provided higher peak currents, lower Δ*E*_p_, low charge-transfer resistance (*R*_ct_), and larger electroactive surface area. In addition, during the proof-of-concept study, a significant increase in the CPS voltammetric response was observed after using the simple and quick treatment. The 3D-CB/PLA–PT sensor proved to be efficient in detecting CPS in pepper sauce samples, with little or no interference from sample matrices. Furthermore, our proposal of treatment has the potential to be used anywhere due to unique characteristics such as low cost, battery-powered, manually operated, portability, and its use only of atmospheric air for plasma generation. To the best of our knowledge, the proposed approach has significant advantages over other previous proposals.

## Supporting Information Available

Experimental section; optimization of surface treatment on the 3D-CB/PLA electrode using a plasma jet pen; reproducibility of electrodes treated with atmospheric air plasma in a redox probe; shelf life study for the 3D-CB/PLA–PT electrode; study of the removal of the surface treatment of the 3D-CB/PLA–PT electrode by means mechanical polishing; performance of the proposed treatment in relation to the chemical/electrochemical treatment; electrochemical performance obtained for the proposed treatment and other activation protocols reported in the literature; performance of the 3D-CB/PLA and 3D-CB/PLA–PT electrodes in dopamine probe; morphological characterization of 3D printing electrodes surface; Raman spectrum and optical images of the 3D-CB/PLA and 3D-CB/PLA–PT electrodes; determination of the double layer capacitance (*C*_dl_) between the printed electrodes; determination of electroactive surface areas; CPS electrochemical behavior study; optimization of the differential pulse voltammetry technique (DPV) parameters; repeatability and reproducibility study of the proposed method using 3D-CB/PLA–PT sensor; surface stability study of the 3D-CB/PLA–PT electrode after measurements in the presence of CPS; proposed treatment in relation to the chemical/electrochemical treatment in the presence of CPS; analytical parameters (linear range and LOD) obtained using 3D-CB/PLA–PT sensor and other sensors reported for the electrochemical determination of CPS; determination of CPS and recovery studies in pepper sauces samples (PDF)
