# Supplementary material for: Portable Atmospheric Air Plasma Jet Pen for the Surface Treatment of Three-Dimensionally (3D)-Printed Electrodes
Source: Anal Chem. 2024 Sep 5;96(40):15852–8. doi: 10.1021/acs.analchem.4c02785 (PMC11465224; doi:10.1021/acs.analchem.4c02785)
Supplement: Supplementary file 1 — ac4c02785_si_001.pdf [file ac4c02785_si_001.pdf]

Supporting Information

Portable atmospheric air plasma jet pen for the surface treatment of 3D-printed electrodes

Gilvana P. Siqueira[a], Raquel G. Rocha[a], Amanda B. Nascimento [a], Eduardo M. Richter[a], Rodrigo A. A. Muñoz[a*]

[a]*Chemistry Institute, Federal University of Uberlândia, 38400-902, Uberlândia, Minas Gerais, Brazil*

Corresponding author:

*munoz@ufu.br *ORCID: 0000-0001-8230-5825*

List of Contents

1. Experimental section……………………………………………………………………….S3
2. Optimization of surface treatment on the 3D-CB/PLA electrode using a plasma jet pen…………S9
3. Reproducibility of electrodes treated with atmospheric air plasma in a redox probe……………S13
4. Shelf-life study for the 3D-CB/PLA-PT electrode…………………………………………...S14
5. Study of the removal of the surface treatment of the 3D-CB/PLA-PT electrode by means mechanical polishing…………………………………………………………………………...S14
6. Performance of the proposed treatment in relation to the chemical/electrochemical treatment…S15
7. Electrochemical performance obtained for the proposed treatment and other activation protocols reported in the literature ………………………………………………………………….S16
8. Performance of the 3D-CB/PLA and 3D-CB/PLA-PT electrodes in dopamine probe………….S17
9. Morphological characterization of 3D printing electrodes surface…………………………….S17
10. Raman spectrum and optical images of the 3D-CB/PLA and 3D-CB/PLA-PT electrodes….…S18
11. Determination of the double layer capacitance (Cdl) between the printed electrodes………….S19

12. Determination of electroactive surface areas……………………………………………………...S20

13. CPS electrochemical behavior study…………………………………………………………S21

14. Optimization of the differential pulse voltammetry technique (DPV) parameters……………...S25

15. Repeatability and reproducibility study of the proposed method using 3D-CB/PLA-PT sensor……………………………………………………………………………………...S26

16. Surface stability study of the 3D-CB/PLA-PT electrode after measurements in the presence of CPS……………………………………………………………………………………….….S28

17. Proposed treatment in relation to the chemical/electrochemical treatment in the presence of CPS………………………………………………………………………………….…….S29

18. Analytical parameters (linear range and LOD) obtained using 3D-CB/PLA-PT sensor and other sensors reported for the electrochemical determination of CPS…………………....................S30

19. Determination of CPS and recovery studies in pepper sauces samples…………………………S32

20. References…………………………………………………………………….….….S37

1. Experimental section

1.1. Reagents and standards

All reagents used in this work were of analytical grade and without further purification. To prepare the aqueous solutions, high-purity deionized water obtained from a Milli Q purification system (Millipore, Bedford, MA, USA) with a resistivity >18 MΩ cm was used. Acetic acid (99.8% w/v) was achieved from Vetec (Rio de Janeiro, Brazil), phosphoric acid (85% w/v) and potassium ferricyanide(III) (99% w/w) were obtained from Labsynth (São Paulo, Brazil). Boric acid (99.8% w/w) and sodium hydroxide (98% w/w) were from AppliChem Panreac (Barcelona, Spain). Ethanol (95% v/v) and potassium chloride (98% w/w) were acquired from EasyPath|Diagnostics (São Paulo, Brazil) and Dinâmica® (São Paulo, Brazil), respectively. Hexaamineruthenium chloride(III) and (II) were obtained (Sigma Aldrich, Steinheim, Germany).

A standard powder containing a mixture of capsaicin (61.7% w/w) and dihydrocapsaicin (32.1% w/w) was obtained from Sigma-Aldrich® (St. Louis, MO, USA). This standard refers the capsaicinoid profile (CPS) on the peppers. CPS stock solutions (20.0 mM), prepared in ethanol, were stored under refrigeration to avoid possible photodegradation processes. Standard solutions of 1.0 mM CPS in appropriate supporting electrolyte were prepared daily and diluted in an electrochemical cell before analysis. Britton-Robinson (BR) buffer (0.12 M) was prepared by mixing 0.04 M of acetic, boric, and phosphoric acids, and adjusting the pH of the solution (2.0 to 12.0) was carried out with NaOH (1.0 M). 0.1 M KCl was added to the buffer solutions to maintain the ionic strength of the solutions.[1]

1.1.1. Sample preparation

Four red pepper sauces produced in different Brazilian regions were chosen as samples for analysis in the electrochemical applications. Samples were purchased from a local supermarket (Uberlândia, Brazil). The samples were subjected to an extraction procedure before electrochemical analysis, according to Deroco *et al*. 2020 [2] with a slight modification. An aliquot of 200 mg of each sample was transferred to a volumetric flask and mixed with 5 mL of ethanol, subsequently, the mixture was sonicated for 60 min and filtered using a 0.45 µm PES membrane syringe filter – K18-430 (KASVI, China); then, this separate solution was diluted (200-fold) in supporting electrolyte in the electrochemical cell and used to quantify the capsainoid profile of hot peppers from these samples. The four pepper sauce samples were called

samples A, B, C and D; samples spiked with 10.0 and 20.0 µM CPS standard solution were named A_{F1}, B_{F1}, C_{F1}, and D_{F1}, and A_{F2}, B_{F2}, C_{F2}, and D_{F2}, respectively.

1.2. 3D printing of electrochemical platforms (electrodes) for subsequent treatment

A commercial filament composed by a mixture of the carbon black and PLA (CB/PLA) (Protopasta®, WA, USA) was used to produce the 3D-printed electrodes. The electrode was printed using an FDM-type printer (Flashforge Dreamer NX, China), and the printing parameters used are described in Table S1.

Table S1. Printing conditions to manufacture the 3D-CB/PLA electrodes.

Printing parameters	Condition
**Orientation**	Horizontal
**Layer weight / mm**	0.05
**Infill density/ %**	100
**Nozzle extruder / mm**	0.6
**Perimeter number / shell**	2
**Printing speed / mm s^{-1}**	70
**Bed temperature/ °C**	90
**Extruder temperature / °C**	210

The design of the working electrode as previously reported in the literature [3]. The working electrode, called 3D-CB/PLA, and their respective dimensions, can be seen in Figure S3A. These electrodes were treated with air plasma, according to the procedure detailed in the next section.

1.3. Treatment of carbon black and PLA electrodes using plasma jet pen

The video of the 3D-CB/PLA-PT electrode surface treatment process can be viewed at the video . Images of the plasma jet pen and the tips used can be found in Figure S1. Schematic representation of how the plasma was applied on the 3D-CB/PLA-PT surface is shown in Figure S2 and Figure S3 displays the

real images of 3D-printed electrodes before (3D-CB/PLA) and after the treatment procedure (3D-CB/PLA-PT).

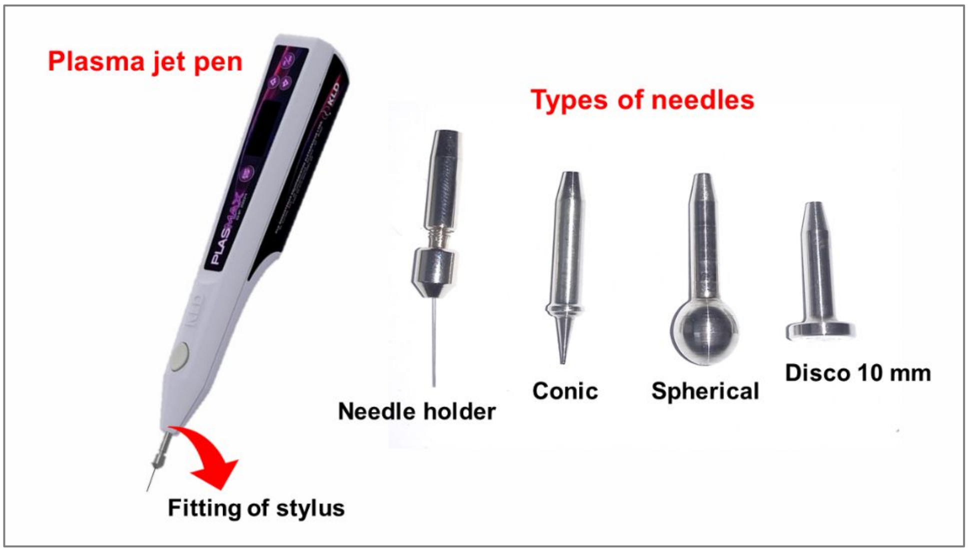

Figure S1. Images of the plasma jet pen and the types of needles used.

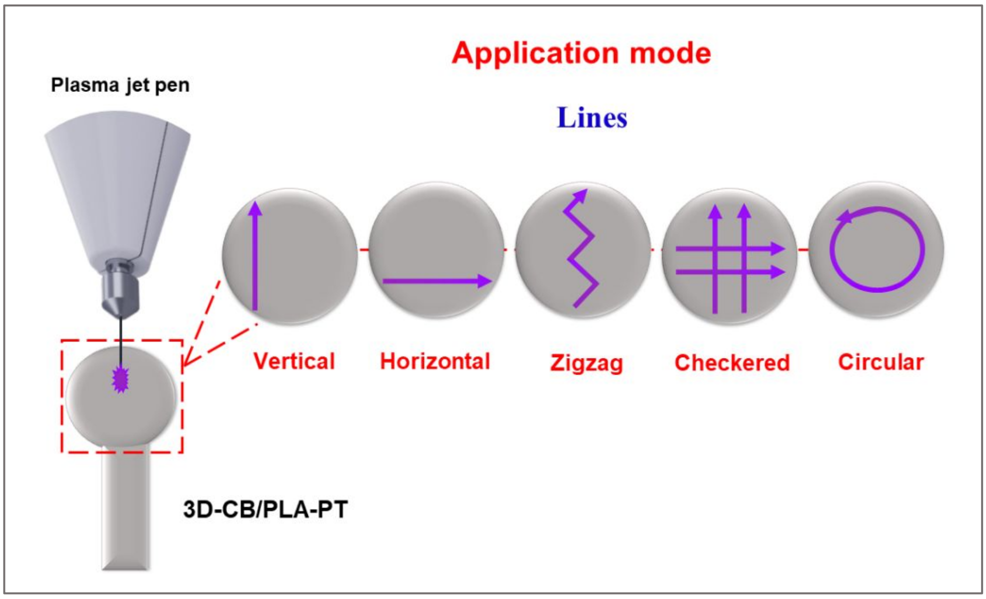

Figure S2. Schematic representation of how the plasma was applied on the 3D-CB/PLA-PT surface.

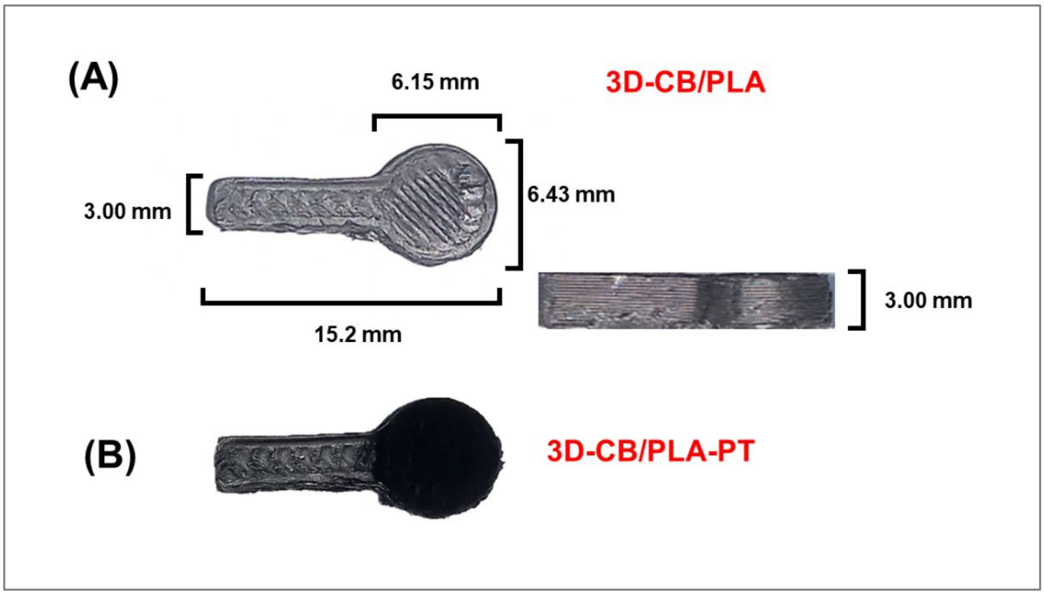

Figure S3. Real image of the **(A)** 3D-CB/PLA and **(B)** 3D-CB/PLA-PT electrodes, and their dimensions. The rectangular piece on the right refers to the thickness of the electrodes.

1.4. Microscopic and spectroscopic characterization of 3D-CB/PLA surfaces

The surface morphology was evaluated using a scanning electron microscope (SEM) Vega 3 microscope (Tescan, Czech Republic), with the electron beam energy of 20.0 kV, controlled by Vega TC software. Raman spectra were acquired on a LabRAM HR Evolution – HORIBA Spectrophotometer (Japan), controlled by software HORIBA Scientific's LabSpec, using a 532 nm laser at 50 mW of power in the range of 4000 to 80 cm^{-1}. Absorption spectra in the Fourier transform infrared (FTIR) region in the ATR MIR/FIR mode (PerkinElmer, USA) with a CsI detector were used to obtain infrared spectra in the region from 200 to 4000 cm^{-1}.

Atomic force microscopy (AFM) images were performed on a Scanning probe microscope (SPM) (SPM-9600, Shimadzu, Japan), obtained in dynamic force mode, using silicon probes PPP-NCHR AFM (NANOSENSORS™, Switzerland) with resonance frequency of 330 kHz, force constant of 42 N/m, length 125 µm, mean width 30 µm and thickness 4µm.

1.5. Apparatus and electrochemical measurements

A 10 mL electrochemical cell was used with three electrodes (3D working electrode, reference ($Ag|AgCl|KCl_{(sat.)}$) and counter electrode (platinum wire)). The electrochemical cell was manufactured

using a GTMax 3D printer (São Paulo, Brazil) with Polyethylene Terephthalate Glycol (PETG) filament (GTMax, São Paulo, Brazil). A rubber O-ring delimited the geometric area of the working electrode (0.23 cm^2). More information about the design, arrangement of electrodes and assembly of the electrochemical cell can be found in previous works by our research group. [4–6]

Cyclic Voltammetry (CV), Differential Pulse Voltammetry (DPV) and electrochemical impedance spectroscopy (EIS) were performed using Autolab PGSTAT204 potentiostat/galvanostat (Metrohm Autolab BV, Utrecht, Netherlands) with EIS FRA32M module, connected to a microcomputer and controlled by NOVA Software 2.1.7.

EIS characterizations were performed using a frequency range between 0.1 Hz and 50 kHz and amplitude of 10 mV, in the presence of 2.0 mM $[Fe(CN)_6]^{3-/4-}$ and 0.1 M KCl, applying a half-wave potential of +0.22 V (*vs* $Ag|AgCl|KCl_{(sat.)}$). The Randles equivalent circuit was applied to acquire the charge transfer resistance (Rct) between the surface of the electrodes and the redox probe.

The double layer capacitance (Cdl) measurements were performed by CV, as described in the literature.[7,8] Cdl values were obtained from the dependence of the curve slope of the peak current density (ΔJ) (ΔJ = difference between the anodic (Ia) and cathodic (Ic) currents divided by the geometric area of the electrodes (0.22 cm^2)) versus scan rate. Current peaks were extracted at a potential of +0.15 V (*vs.* $Ag|AgCl|KCl_{(sat.)}$) in a potential window of 0 to +0.3 V (*vs.* $Ag|AgCl|KCl_{(sat.)}$) in 0.1 M KCl, by CV, under increasing scan rates (10 to 30 mV s^{-1}).

The electrochemically active surface area of the 3D-CB/PLA and 3D-CB/PLA-PT electrodes was estimated through CV experiments conducted at various scan rates (0.01 to 0.2 V s^{-1}) using the 1:1 mM $[Ru(NH_3)_6]^{2+/3+}$ probe, following the Randles-Sevcik equation 1.[9,10]

$$I_p = 2.69 \times 10^5 n^{3/2} A_{real} C \sqrt{Dv} \quad (1)$$

Where I_p is the peak current, n is the number of electrons transferred (n = 1, for $[Ru(NH_3)_6]^{2+/3+}$ probe), A_{real} is the electroactive area (cm^2), D is the diffusion coefficient of the species (D = 8.43×10^{-6} cm^2 s^{-1}, for hexaammineruthenium(III) cation [11], v is the scan rate (V s^{-1}), C is the bulk concentration of the electroactive species (mol cm^{-3}). The constant with value 2.69×10^5 has units of C mol^{-1} $V^{-1/2}$.

In the study of shelf-life stability of the treated sensor, tests were conducted on the same 3D-CB/PLA-PT electrode over several days in the presence of 2 mM $[Fe(CN)_6]^{3-/4-}$ by CV measurements. The electrode was stored at room temperature and in a closed compartment.

In the stability study of the 3D-CB/PLA-PT sensor, over 100 measurements were conducted in the presence of CPS in a single day. Additionally, measurements were taken on the $[Fe(CN)_6]^{3-/4-}$ probe before and after the CPS measurements.

The electrodes mechanically treated were named as 3D-CB/PLA-MT. Prior to use, they were polished on sandpaper (600 grit followed by 1200 grit, obtained from local stores), for 30 seconds each, using ultrapure water.

The 3D-CB/PLA electrodes subjected to chemical and electrochemical treatment were designated as 3D-CB/PLA-QET, following the method established by Richter *et al.* 2019 [12]. Initially, the electrodes were polished on sandpaper for 30 s each (600 grit followed by 1200 grit) using ultrapure water. Subsequently, the electrodes underwent electrochemical activation (+1.4 V for 200 s and -1.0 V for 200 s in 0.5 M NaOH solution).

The limit of detection (LOD) and limit of quantification (LOQ) values were estimated according to IUPAC recommendations [13], namely: LOD = 3 × SD/S and LOQ = 10 × SD /S where SD is the standard deviation of noise from 10 measurements without the analyte (blank) and S is the slope of the calibration curve.

2. Optimization of surface treatment on the 3D-CB/PLA electrode using a plasma jet pen

The electrochemical performance of the 3D printed electrodes, before and after atmospheric air plasma treatment, was evaluated regarding the reversibility of the redox couple $[Fe(CN)_6]^{3-/4-}$ by CV measurements. Figure S4 (A-D) shows the cyclic voltammograms of 3D-CB/PLA-PT electrode, varying the generation plasma and surface treatment conditions. The results were compared following two criteria: the peak-to-peak separation (ΔEp), and the ratio between the anodic (Ia) and the cathodic peak (Ic) currents. The obtained values are given in Table S2.

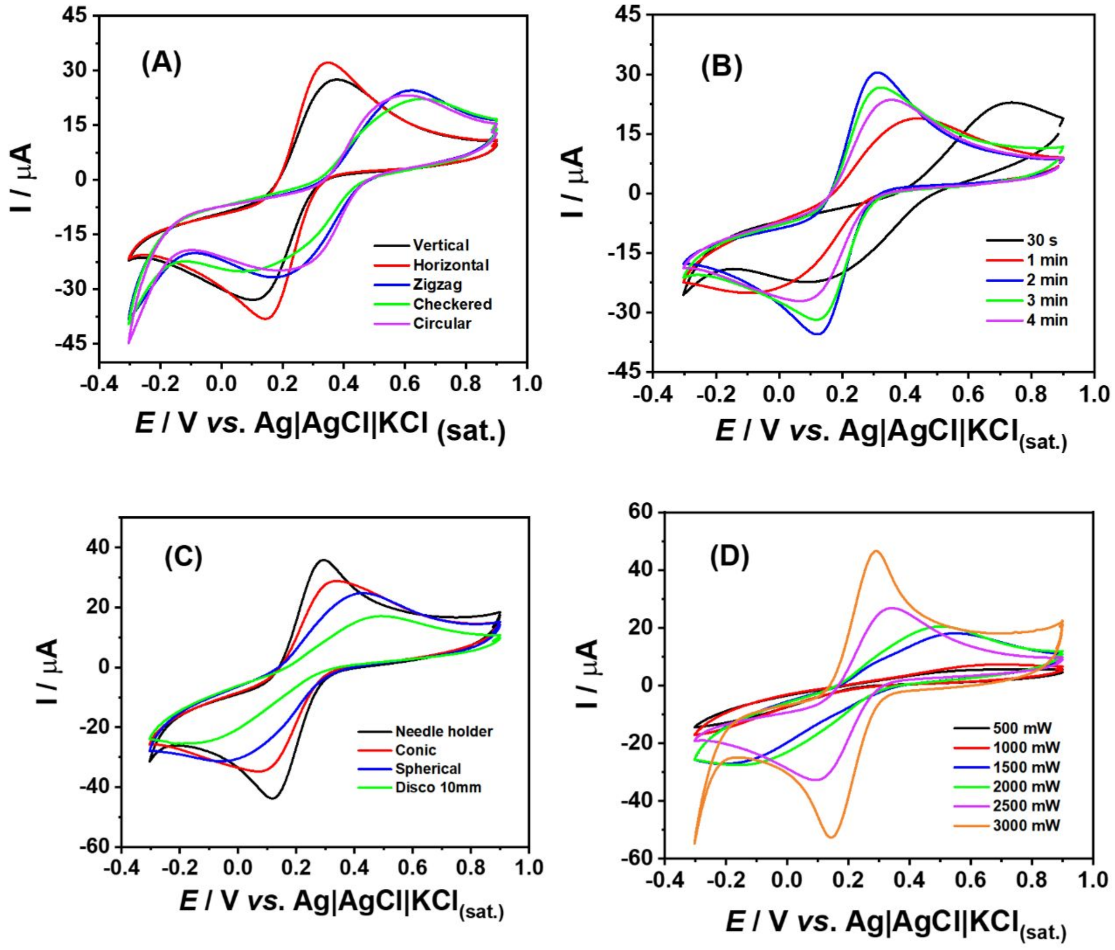

Figure S4. Effect of application modes **(A)** and time **(B)**, type of needles **(C)** and plasma power **(D)** in the cyclic voltametric response of 2 mM $[Fe(CN)6]^{3-/4-}$ in 0.1 M KCl solution using 3D-CB/PLA-PT working electrode. **CV conditions:** scan rate = 50 mV s^{-1}; step potential = -5 mV.

Table S2. Studied parameters of atmospheric air plasma treatment.

Parameters		ΔEp /mV	Ipa/Ipc
**Application mode**	Vertical	240±3	1.126±0.007
	Horizontal	188±3	1.063±0.009
	Zigzag;	376±8	1.006±0.018
	Checkered	440±10	1.116±0.039
	Circular	299 ±6	1.013±0.030
**Application time**	30s	512±21	0.884±0.006
	1min	396±3	1.499±0.005
	2min	176±1	1.109±0.002
	3min	182±1	1.128±0.003
	4min	245±3	1.261±0.004
**Types of needles**	Needle holder	165±3	1.090±0.004
	Conic	225±3	1.181±0.012
	Spherical	371±13	1.414±0.021
	Disco 10mm	514±5	1.606±0.006
**Plasma power (mW)**	500	*	*
	1000	*	*
	1500	616±13	1.884±0.087
	2000	522±3	1.699±0.014
	2500	223±3	1.112±0.002
	3000	143±2	1.082±0.004

* Using powers of 500 and 1000 mW, there was no definition of the cathodic current peak, so it was not possible to calculate these relationships.

Using the plasma jet pen in generation continuous mode, with less than 1 mm between the tip of the plasma jet pen and the surface of the 3D-CB/PLA-PT electrode, different ways of applying plasma to the electrode surface were evaluated, as shown in Figure S4A. The application modes (lines: Vertical, Horizontal, Zigzag, Checkered and Circular) significantly improve the electrochemical performance. As can be seen, better voltammetric profile and greater faradaic current response were achieved when plasma treatment was applied with vertical and horizontal line orientation, as observed in the Figure S4A. However, in terms of ΔEp and Ipa/Ipc, the treatment in horizontal lines exhibited better reversibility (ΔEp = 188 mV and Ipa/Ipc = 1.06) than the treatment in vertical lines (ΔEp = 240 mV and Ipa/Ipc = 1.27), in Table S2. Thus, we selected the application mode in horizontal orientation.

Once the plasma application orientation was fixed in horizontal lines, the application time was evaluated (30 s to 4 min), see Figure S4B. In times less than 2 min, ill-defined cathodic and anodic peaks were achieved with higher ΔEp values, as seen in Table S2. However, when treatment time was fixed at 2 min, a lower ΔEp was acquired between the times tested (ΔEp = 176 mV) and Ipa/Ipc very close to unity (Ipa/Ipc = 1.11), in longer times (3 and 4 min), even with high values of anodic and cathodic current, behavior that is far from to ideal distant would be obtained and the required time to modify each electrode would be higher.

The penultimate optimized condition refers to the type of needles to be used during plasma application. The tips are available from the manufacturer in different formats (needle holder, conical, spherical, disco 10 mm) and they are attached in the plasma jet pen, as shown in Figure S4C. From the voltammetric results, the tip of the needle holder showed a better electrochemical response, with higher anodic and cathodic current values, and better reversibility in the probe (ΔEp = 165 mV and Ipa/Ipc = 1.09), which can be explained by the greater concentration of plasma energy at the smaller end.

Finally, the last optimized condition was the plasma power of the plasma jet pen, varying the powers from 500 to 3000 mW as can be noticed in the Figure S4D. Using powers of 500 and 1000 mW, exhibited poorly defined faradaic peaks (only a low oxidation intensity peaks (Ipa = 2.53 µA and 3.27µA, respectively) are obtained for the probe). This behavior is far from the expected for the $[Fe(CN)_6]^{3-/4-}$ probe on carbonaceous surfaces. Probably, the applied energy in these conditions is not sufficient to remove the PLA and expose more conductive sites on 3D-printed CB/PLA surface. Nevertheless, when powers of 1500 and 2000 mW were applied, the presence of reduction and oxidation peaks is noted with, a significant

improve when compared to the results at lower powers. The best behaviors were observed using powers of 2500 and 3000 mW. The anodic and cathodic current values for 3000 mW were higher, as well the peak-to-peak separation and the ratio between anodic and cathodic peaks (ΔEp = 143 mV and Ipa/Ipc = 1.08) when compared to the application of 2500 mW (ΔEp = 223 mV and Ipa/Ipc = 1.12). Table S3 summarizes the studied parameters and optimized condition selected for the air plasma jet pen treatment.

Table S3. The range plasma generation and optimized conditions for surface treatment of 3D-CB/PLA-PT electrodes using atmospheric air plasma jet pen.

Plasma jet pen treatment	Conditions	Optimized
**Plasma generation mode**	Continuous	-
**Distance between the types of needles of the plasma jet pen and the electrode surface**	<1mm	-
**Plasma power**	500 – 3000 mW	3000
**Types of needles**	Needle holder; Conic; Spherical; Disco 10mm	Needle holder
**Aplication mode**	Lines (Vertical; Horizontal; Zigzag; Checkered; Circular)	Horizontal lines
**Application time**	30 s - 4 min	2 min

3. Reproducibility of electrodes treated with atmospheric air plasma in a redox probe

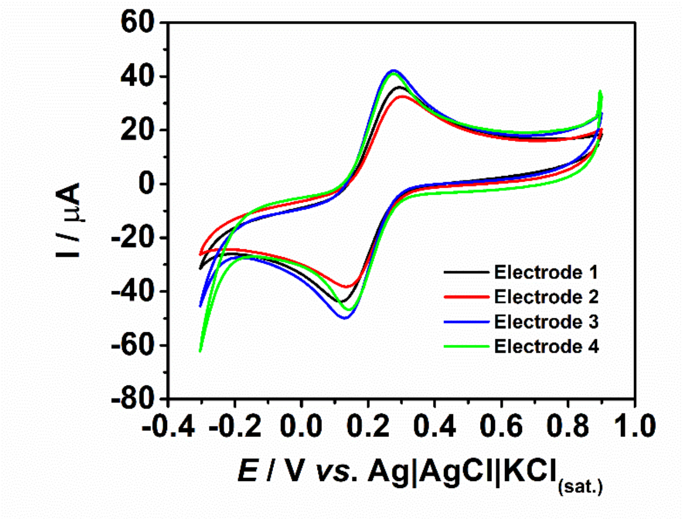

Figure S5. Reproducibility study (n=4) for the 3D-CB/PLA-PT electrode, under optimized conditions of treatment with atmospheric air plasma, at the redox couple 2 mM $[Fe(CN)_6]^{3-/4-}$ in 0.1 M of KCl by CV. **CV conditions:** scan rate = 50 mV s^{-1}; step potential = -5 mV.

Table S4. Peak-to-peak separation values (ΔEp) and relationship between anodic current and cathodic peak current (Ipa/Ipc) of four 3D-CB/PLA-PT electrodes, under optimized conditions of atmospheric air plasma treatment. Responses were obtained for the redox couple 2 mM $[Fe(CN)_6]^{3-/4-}$ in 0.1 M of KCl solution by CV.

Electrodes	ΔEp /mV	Ipa/Ipc
**1**	134±3	1.075±0.008
**2**	129±3	1.110±0.009
**3**	162±3	1.098±0.011
**4**	151±1	1.076±0.008

4. Shelf-life study for the 3D-CB/PLA-PT electrode

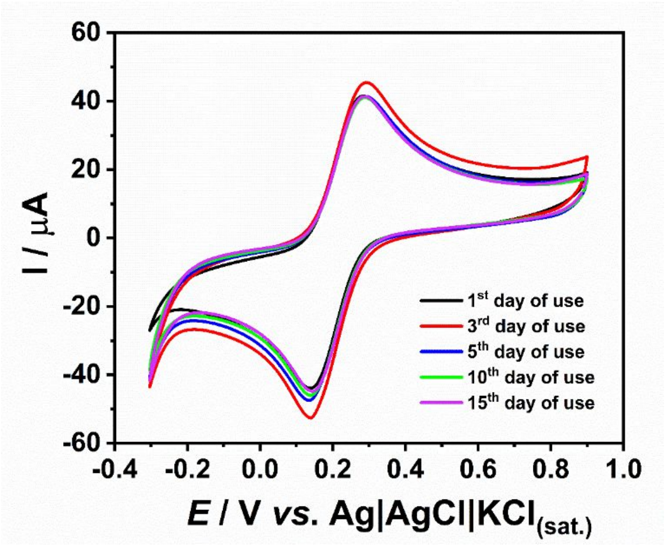

Figure S6. Shelf-life study for the 3D-CB/PLA-PT electrode, at the redox couple 2 mM $[Fe(CN)_6]^{3-/4-}$ in 0.1 M of KCl by CV measurements. **CV conditions**: scan rate = 50 mV s^{-1}; step potential = -5 mV.

5. Study of the removal of the surface treatment of the 3D-CB/PLA-PT electrode by mechanical polishing

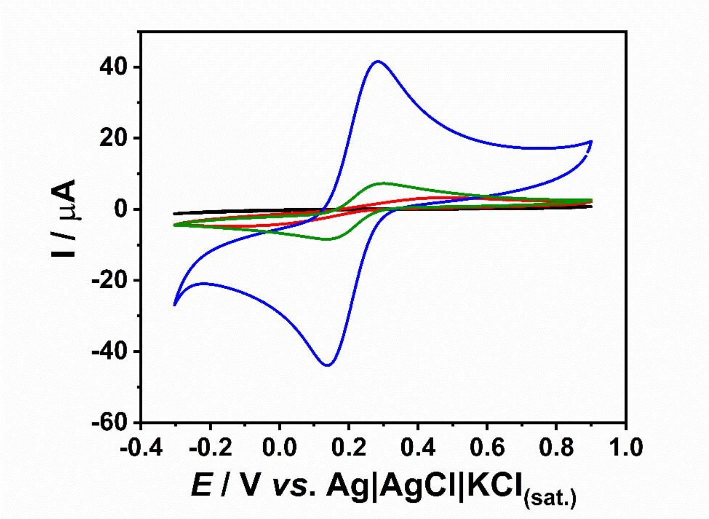

Figure S7. Study of the removal of 3D-CB/PLA-PT electrode surface treatment performing mechanical polishing, in the presence of 2 mM $[Fe(CN)_6]^{3-/4-}$ in 0.1 M of KCl by CV; untreated electrode (3D-CB/PLA, black line), electrodes treated mechanically (3D-CB/PLA-MT, red line), electrode treated with

atmospheric air plasma (3D-CB/PLA-PT, blue line), and mechanical treatment on electrode after atmospheric air plasma treatment (green line). **CV conditions**: scan rate = 50 mV s^{-1}; step potential = -5 mV.

6. Performance of the proposed treatment in relation to the chemical/electrochemical treatment

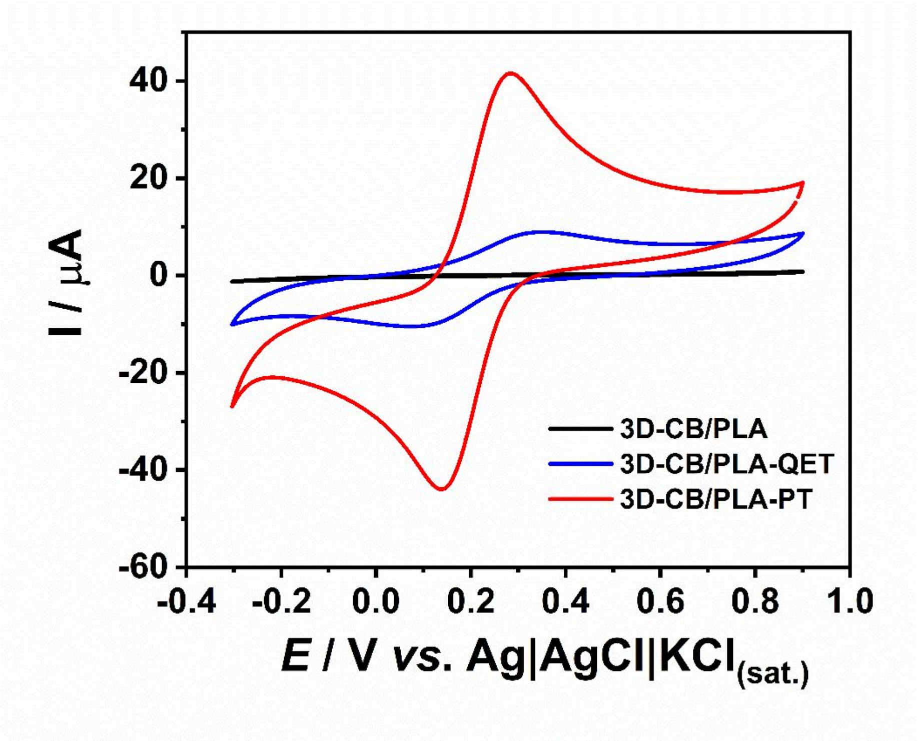

Figure S8. CV measurements on 3D-CB/PLA (black line), 3D-CB/PLA-QET (blue line) and 3D-CB/PLA-PT (red line) in the presence at the redox couple 2 mM $[Fe(CN)_6]^{3-/4-}$ in 0.1 M of KCl. **CV conditions**: scan rate = 50 mV s^{-1}; step potential = -5 mV.

7. Electrochemical performance obtained for the proposed treatment and other activation protocols reported in the literature

Table S5. Comparison of the electrochemical key parameters obtained from electrochemical characterization of the proposed atmospheric air plasma surface treatment with other treatment protocols reported in the literature.

Treatment method	Electrode	Electrochemical redox probe	ΔE_p (V)	Ipa/Ipc	Time	Ref.
**Electrochemical**	PLA/Gr/GO	$[Fe(CN)_6]^{3-/4-}$	0.370	-	-	[14]
**Chemical**	nC/PLA	$[Fe(CN)_6]^{3-/4-}$	0.450	-	24 hours	[15]
**Reactive oxygen plasma**	CB/PLA	$[Fe(CN)_6]^{3-/4-}$	0.156	1.10	2 min.	[16]
**Physical thermal annealing**	G/PLA	$[Fe(CN)_6]^{3-/4-}$	0.255	-	4.3 hours	[17]
**Solvent/electrochemical**	G/PLA	$[Fe(CN)_6]^{3-/4-}$	0.171	-	> 24 hours	[18]
**Enzymatic digestion**	G/PLA	$[Fe(CN)_6]^{3-/4-}$	0.180	-	33 min.	[19]
**Electrochemical**	G/PLA	$[Fe(CN)_6]^{3-/4-}$	0.180	-	30 min.	[20]
**Laser-scribing**	CB/PLA	$[Fe(CN)_6]^{3-/4-}$	0.130	1.12	< 1 min.	[21]
**Laser-ablation**	CB/PLA	$[Ru(NH_3)_6]^{3+/2+}$	0.161	0.88	-	[22]
**Chemical/electrochemical**	CB/PLA	$[Fe(CN)_6]^{3-/4-}$	0.297	-	6.7 min.	[23]
**Laser-ablation/electrochemical**	CB/PLA	$[Fe(CN)_6]^{3-/4-}$	0.190	1	7.5 min.	[24]
**Atmospheric air plasma**	CB/PLA	$[Fe(CN)_6]^{3-/4-}$	0.134	1.09	2 min.	This work

PLA/Gr/GO: electrode comprised of polylactic acid (PLA) and graphite (Gr) doped with graphene oxide (GO); **nC/PLA**: nanocarbon and polylactic acid electrode; **CB/PLA**: carbon black and polylactic acid electrode; **G/PLA**: graphene and polylactic acid electrode.

8. Performance of the 3D-CB/PLA and 3D-CB/PLA-PT electrodes in dopamine probe

Table S6. Peak-to-peak separation values (ΔEp) and relationship between anodic and cathodic peak current (Ipa/Ipc) for 3D-CB/PLA and 3D-CB/PLA-PT electrodes in the presence of 1 mM dopamine in 0.1 M $HClO_4$ solution by CV measurements.

Electrodes	ΔEp /mV	Ipa/Ipc
**3D-CB/PLA**	814±3	2.122±0.115
**3D-CB/PLA-PT**	191±10	1.095±0.079

9. Morphological characterization of 3D printing electrode surfaces

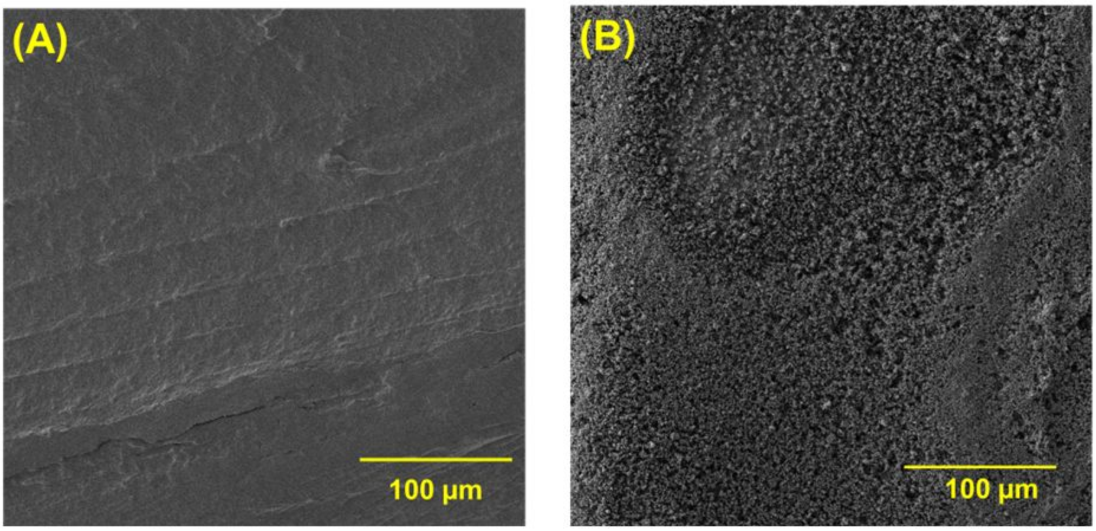

Figure S9. SEM images at lower magnifications of the untreated (3D-CB/PLA) **(A)** and atmospheric air plasma treated (3D-CB/PLA-PT) **(B)** electrodes.

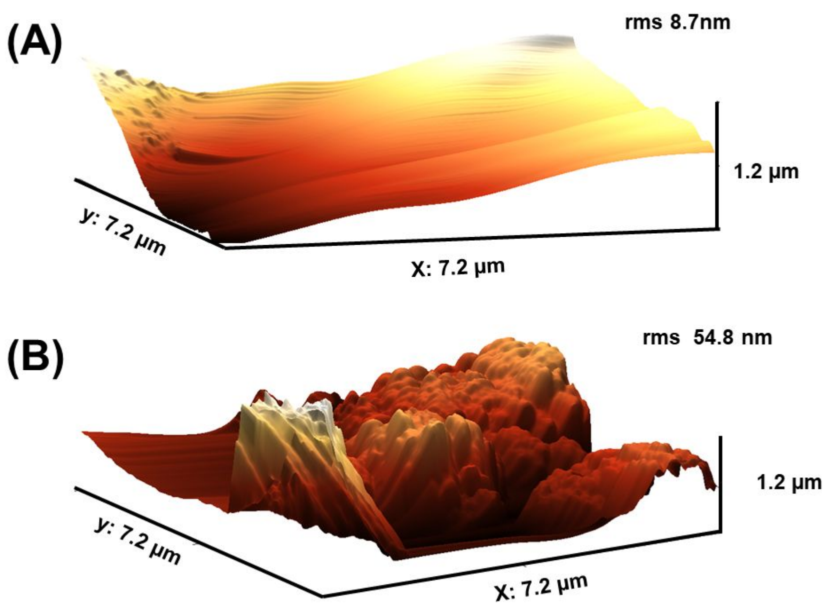

Figure S10. AFM images of the surface of both **(A)** 3D-CB/PLA and **(B)** 3D-CB/PLA-PT electrodes.

10. Raman spectrum and optical images of the 3D-CB/PLA and 3D-CB/PLA-PT electrodes

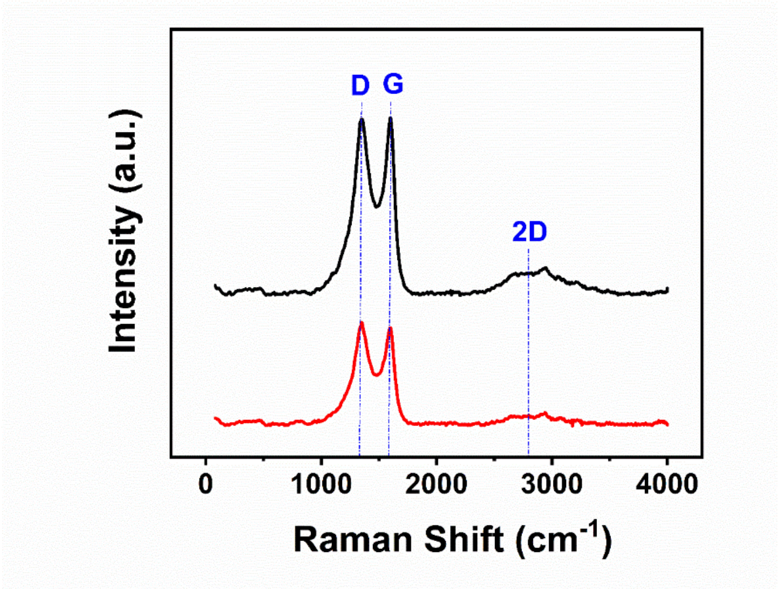

Figure S11. Raman spectra obtained for the 3D-CB/PLA (black line) and 3D-CB/PLA-PT (red line) electrodes surface.

11. Determination of the double layer capacitance (Cdl) between the printed electrodes

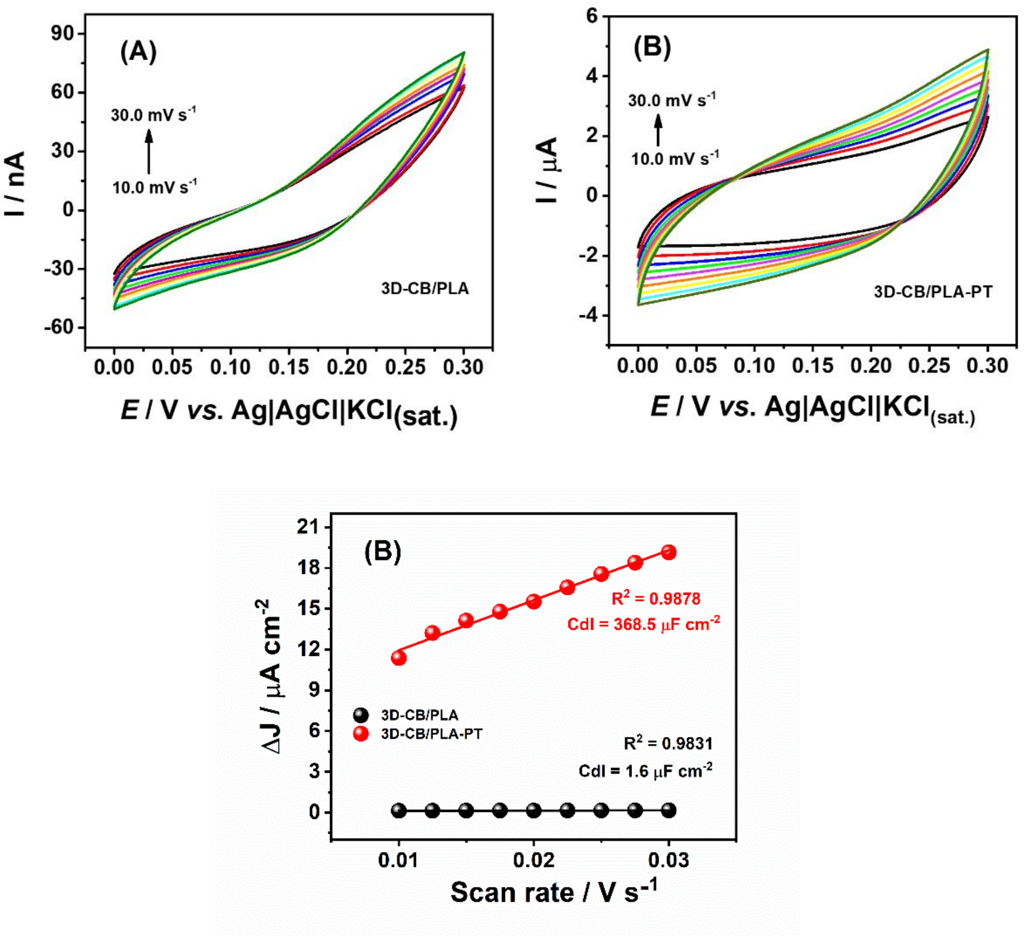

Figure S12. CV recorded at 10 to 30 mV s^{-1} at the **(A)** 3D-CB/PLA and **(B)** 3D-CB/PLA-PT electrodes. CVs were measured in 0.1 M KCl solution from 0 to +0.3 V *vs*. (Ag|AgCl|$KCl_{(sat.)}$). **(C)** Capacitance data: plots of ΔJ (peak currents were measured at +0.15 V (*vs*. Ag|AgCl|$KCl_{(sat.)}$) versus CV scan rate on 3D-CB/PLA and 3D-CB/PLA-PT electrodes to determine the Cdl value

12. Determination of electroactive surface areas

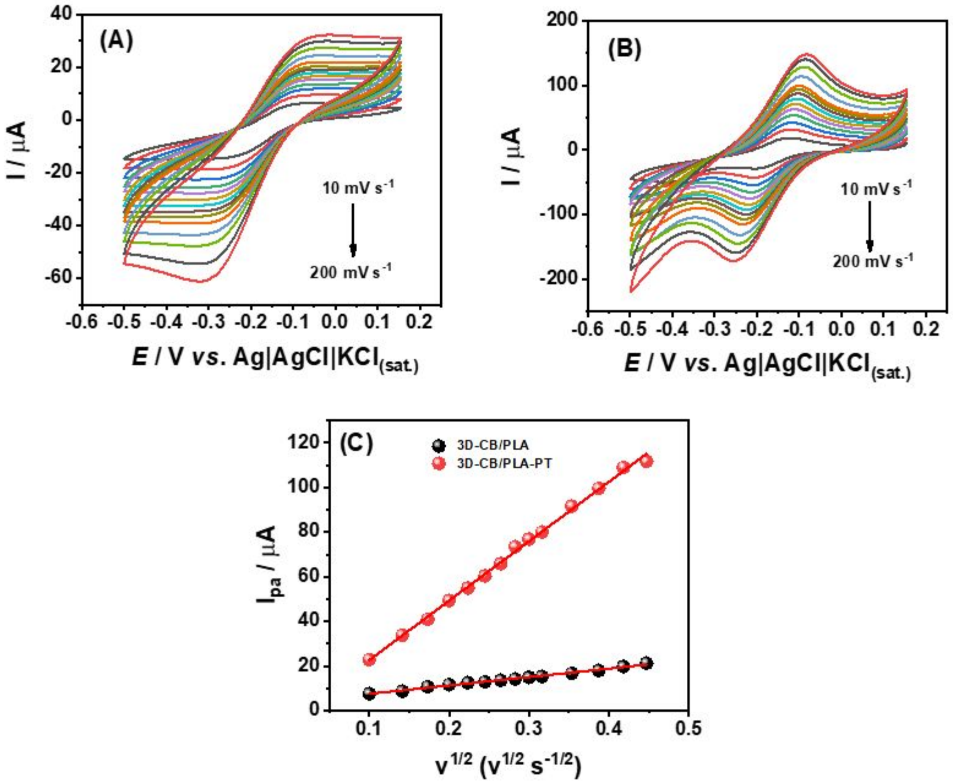

Figure S13. CV recorded at 0.01 to 0.2 V s^{-1} at the **(A)** 3D-CB/PLA and **(B)** 3D-CB/PLA-PT electrodes. CVs were measured in the presence of 1:1 mM $[Ru(NH_3)_6]^{2+/3+}$ in 0.1 M KCl, and **(C)** plot of anodic peak currents (Ipa) versus square root of scan rate ($v^{1/2}$).

Linear regression equations:

$I_{pa}(\mu A) = 37.7\ (\pm 9.9)\ v^{1/2}(V^{1/2}\ s^{-1/2}) + 3.76\ (\pm 2.89)$ $R^2 = 0.992$, for the 3D-CB/PLA electrode

$I_{pa}(\mu A) = 266.1\ (\pm 3.8)\ v^{1/2}(V^{1/2}\ s^{-1/2}) - 3.93\ (\pm 1.12)$ $R^2 = 0.998$, for the 3D-CB/PLA-PT electrode

13. CPS electrochemical behavior study

The electrochemical behavior of capsaicinoid profile (CPS) was studied by CV using the proposed 3D-CB/PLA-PT sensor and its response was compared with the untreated 3D-CB/PLA electrode. In Figure 14, three consecutive cyclic voltammograms obtained for 0.1 mM CPS in 0.12 M BR buffer solution is presented. As can be seen, the first scan using 3D-CB/PLA-PT as working electrode (see Figure 14A), carried out in the anodic direction, presented an oxidation peak (I) at +0.708 V and a reduction peak (II) at +0.406 V. In the second scan, the intensity of peak I significantly decreased, and another oxidation peak (III) appears at +0.486 V. On the other hand, when the untreated 3D-CB/PLA electrode was used as working electrode (Figure 14B), it is also possible to observe the CPS peak redox potentials (peak I, Ep = +0.823 V; peak II, Ep = +0.154 V; peak III, Ep = +0.446), with poorly voltametric profile and intensities of peaks current in the 3D-CB/PLA electrode. It is worth highlighting, reduction peak intensity increases 28-fold using the 3D-CB/PLA-PT electrode when compared to the untreated 3D-CB/PLA electrode. The dependence between electrochemical processes was also investigated in wider potential (+1.0 to -0.4 V) narrower (+0.65 to +0.15 V) (Figure S15A). The reduction peak appears in the cathodic direction, demonstrating that it is independent of the oxidation process. Furthermore, it is possible to observe that the peak at +0.486 V, it is only formed after reduction process, thus forming a redox couple (peak II and III). This was proven by the reduction of the potential window, in which the redox couple is clearly present, even the electrochemical process at +0.708 V did not occurs (Figure S15B). The formation of redox couple occurs due to the formation of the reduction product (catechol), which is oxidized more easily than CPS, as less potential is required. More details about the oxidation and reduction mechanism of CPS can be found in previous works in the literature.[2,25] In addition, the ΔEp obtained for the reversible redox couple (peak II and III) using the 3D-CB/PLA-PT electrode (ΔEp = 94 mV) reduced 300% when compared to the 3D-CB/PLA electrode (ΔEp = 292 mV), demonstrating the electrocatalytic effect of atmospheric air plasma jet pen treatment. Therefore, subsequent experiments were carried out using the 3D-CB/PLA-PT sensor.

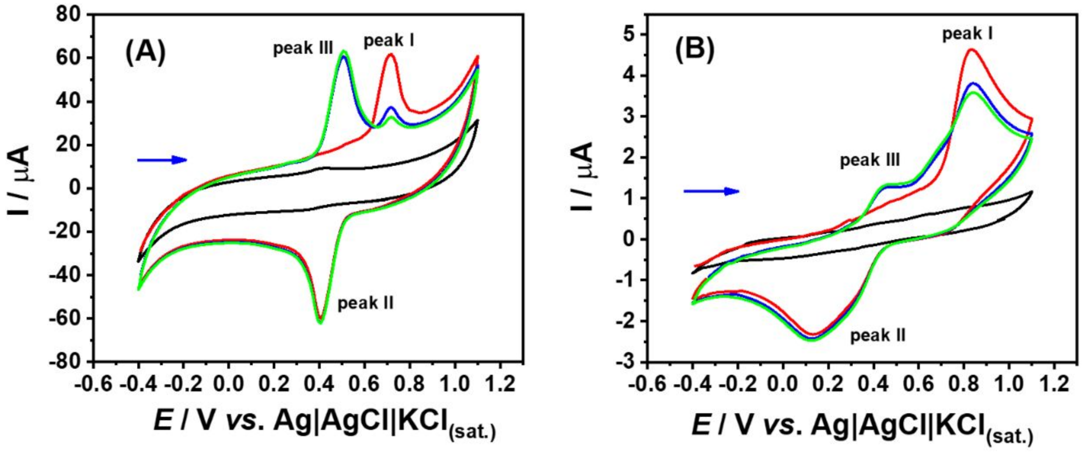

Figure S14. The first (red line), second (blue line) and third (green line) scans for 100.0 µM CPS on the **(A)** 3D-CB/PLA-PT and **(B)** 3D-CB/PLA electrodes in 0.12 M BR buffer solution (pH 2.0). The black lines refer to the blank signal. **CV conditions:** scan rate = 100 mV s^{-1}; step potential = 10 mV.

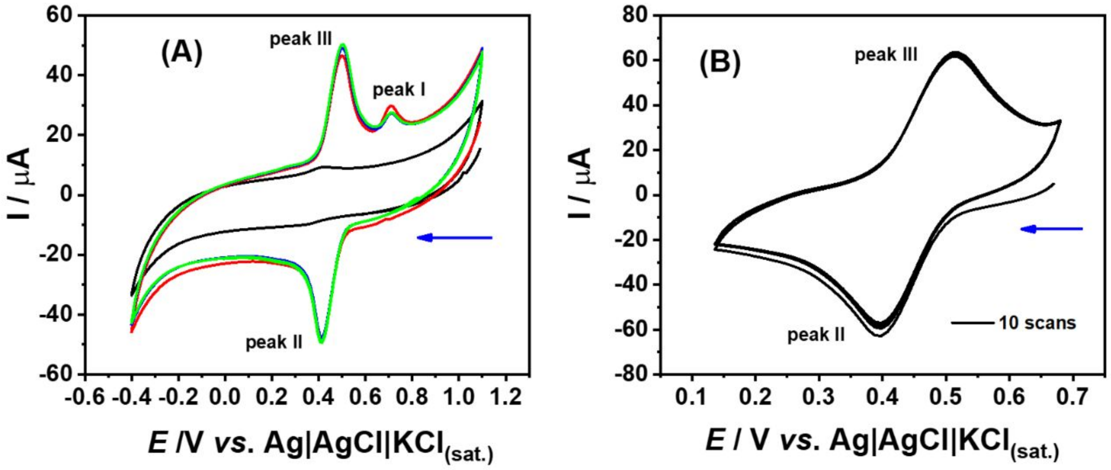

Figure S15. **(A)** The first (red line), second (blue line) and third (green line) scans for 100.0 µM CPS on the 3D-CB/PLA-PT electrode in 0.12 M BR buffer solution (pH 2.0). The black lines refer to the blank signal. **(B)** CV plot restricting of the potential window for the reversible redox couple. **CV conditions:** scan rate = 100 mV s^{-1}; step potential = -10 mV.

In next step, the influence of the pH on the electrochemical behavior of 0.1 mM CPS was evaluated by CV using 0.12 M BR buffer + 0.1 M KCl solution with pH values ranging from 2.0 to 12.0. Figure S16A showed the 1st scan and Figure S16B and C presented the pH influence in the current intensity and peak position for oxidation (peak I) and reduction (peak II) processes. As noticed, at pH values higher than 8.0, the resolution of peaks and the peak current intensity decreased. Linear relationships between peak

potentials and pH, and slope values of 53.6 and 52.4 mV pH^{-1} were assessed for peak I and II, respectively, in which are close to the theoretical value of 59.2 mV pH^{-1} (25 °C) based on Nernst equation, suggesting the same number of protons and electrons in the redox reactions. These values are agreed with previous works ($2H^+$ and $2e^-$).[2,25–27] In the 0.12 M BR buffer (pH 2.0), higher peak intensities were obtained compared to other pH values and a smaller peak half-height width. Therefore, 0.12 M BR buffer (pH 2.0) was chosen as the supporting electrolyte for subsequent measurements. Furthermore, due to the stability of peak II on the surface of the 3D-CB/PLA-PT electrode, it was used for the detections and quantifications in this work.

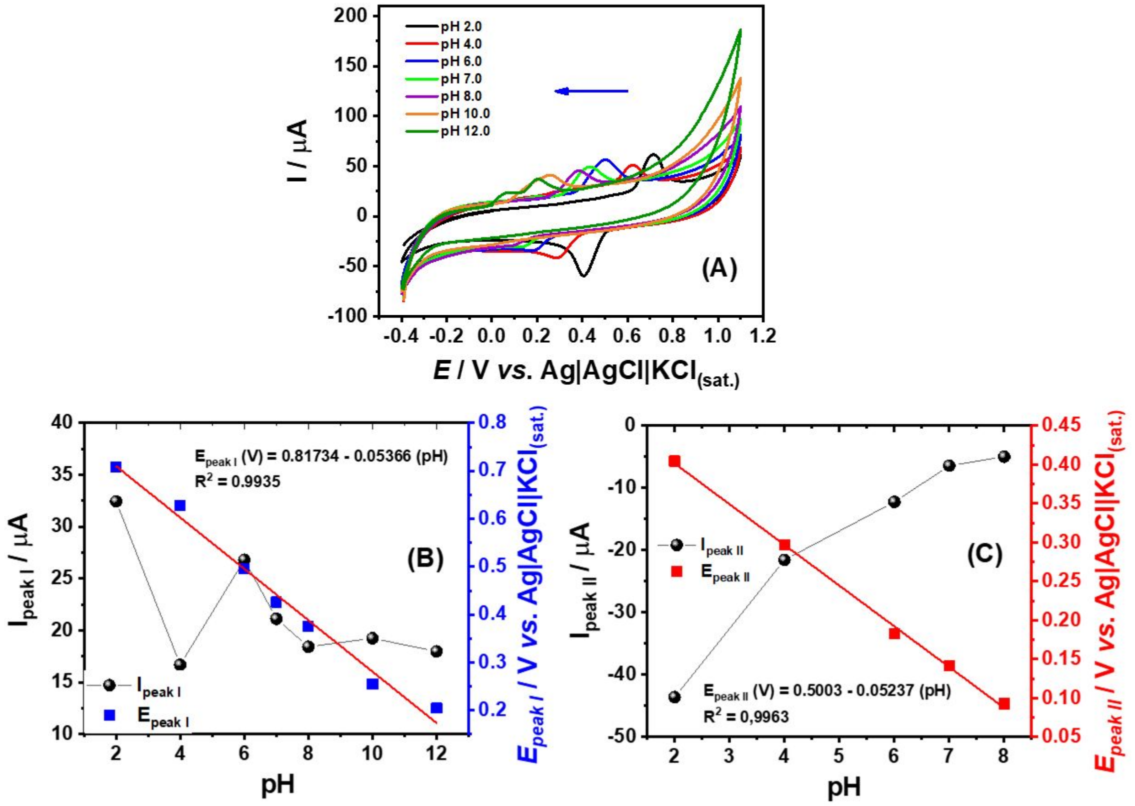

Figure S16. (A) Effect of pH (2.0 – 12.0) on the electrochemical response for 100.0 μM CPS using 0.12 M BR buffer solution; **(B)** Relation of oxidation ($I_{peak\ I}$) and peak potential ($E_{peak\ I}$) versus pH, and **(C)** Relation of reduction process ($I_{peak\ II}$) and peak potential ($E_{peak\ II}$) versus pH. **CV conditions:** scan rate = 100 mV s^{-1}; step potential = 10 mV.

The effect of scan rate on CPS oxidation (peak III) and reduction (peak II) processes currents was studied for the reversible redox couple using the 3D-CB/PLA-PT working electrode in 0.12 M BR buffer (pH 2.0). Figure S17A shows the cyclic voltammetric signals of the 100.0 μM CPS in the range of 10 to 100 mV s^{-1}. The relationship between $I_{peak\ II}$ and $I_{peak\ III}$ *vs*. ν (R^2 = 0.923 and 0.959, respectively, Figure S17B) and $I_{peak\ II}$ and $I_{peak\ III}$ *vs*. $\nu^{1/2}$ (R^2 = 0.985 and 0.998, respectively, Figure S17C), confirms that the kinetics of CPS species in solution is under the control of diffusion.[28]

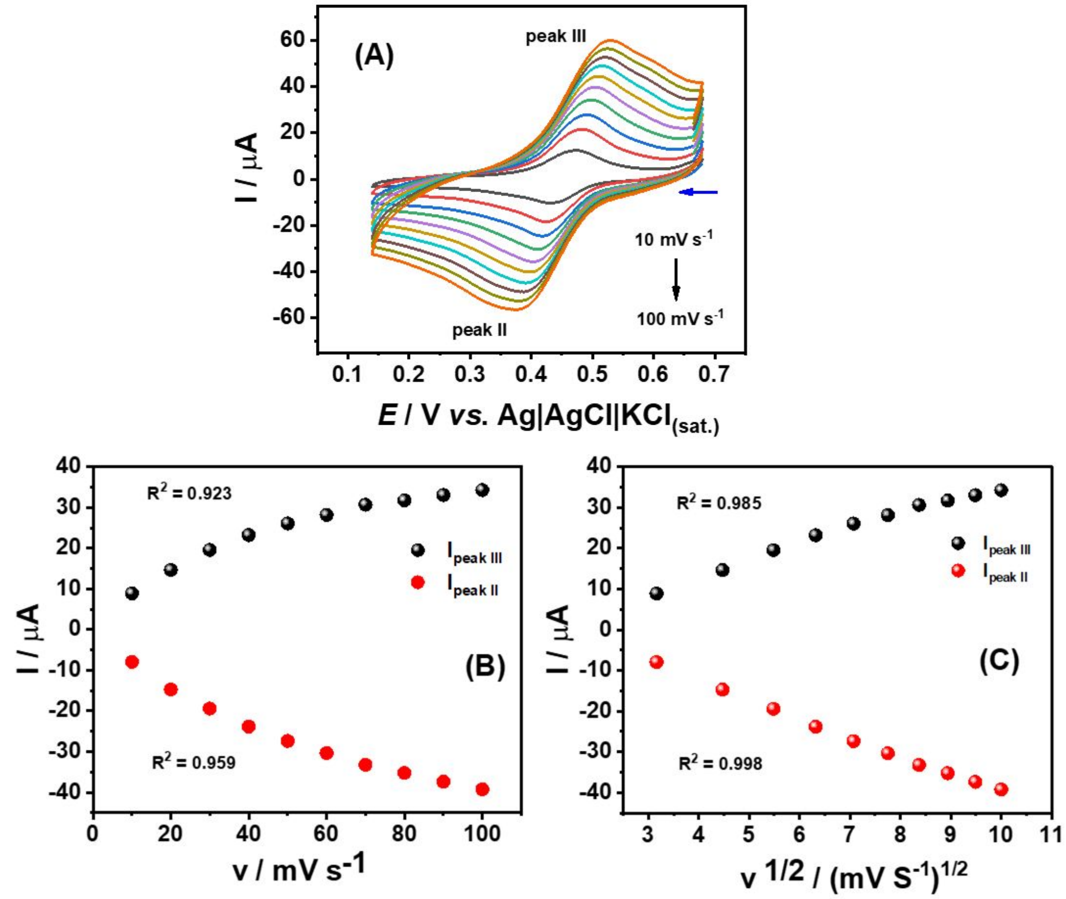

Figure S17. **(A)** Scan rate dependence (10 to 100 mV s^{-1}) in the oxidation (peak III) and reduction (peak II) peak currents of cyclic voltammetry of 100.0 μM CPS on the 3D-CB/PLA-PT electrode in 0.12 M BR buffer (pH 2.0); **(B)** Plot of peak current versus scan rate or **(C)** square root of scan rate.

14. Optimization of the differential pulse voltammetry technique (DPV) parameters

The electrochemical detection of CPS on the 3D-CB/PLA-PT electrode was studied by differential pulse voltammetry (DPV). In the presence of 4 μM CPS, the parameters of the DPV technique were optimized, considering the peak shape (peak half-height width), the intensity current (sensitivity) of the reduction processes (peak II), and the standard deviation between consecutive measurements (n = 3). Figures S18A to S18F display the results obtained for each parameter studied: amplitude (10 to 100 mV), step potential (-1 to -10 mV) and modulation time (10 to 60 ms); from which the ideal parameters were selected (amplitude of 80 mV, step potential of -6 mV and modulation time of 30 ms), highlighted in Table S7.

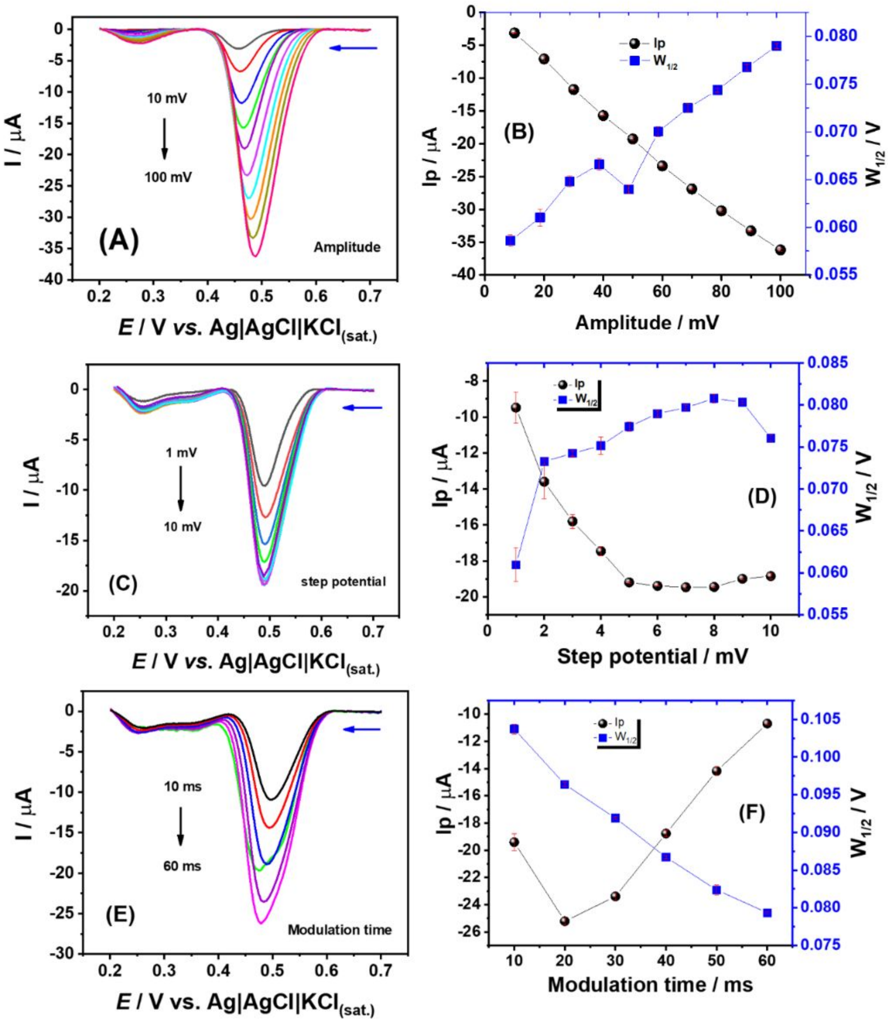

Figura S18. Effect of amplitude (**A:** 10 – 100 mV), step potential (**C:** 1 – 10 mV), and modulation time (**E:** 10 – 60 ms) on the CPS DPV response. Graphs **B**, **D** and **F** refer to the values of peak current (Ip), peak half-height width ($W_{1/2}$) versus each studied parameter of the DPV technique. The optimizations were performed using 0.12 M BR buffer (pH 2.0) as supporting electrolyte containing 4.0 μM CPS.

Table S7: Studied ranges and selected values for the determination of CPS using DPV.

Parameters	Studied range	Selected value
**Amplitude / mV**	10 - 100	80
**Step/ mV**	-1 to -10	-6
**Modulation Time / ms**	10 - 60	30

15. Repeatability and reproducibility study of the proposed method using 3D-CB/PLA-PT sensor

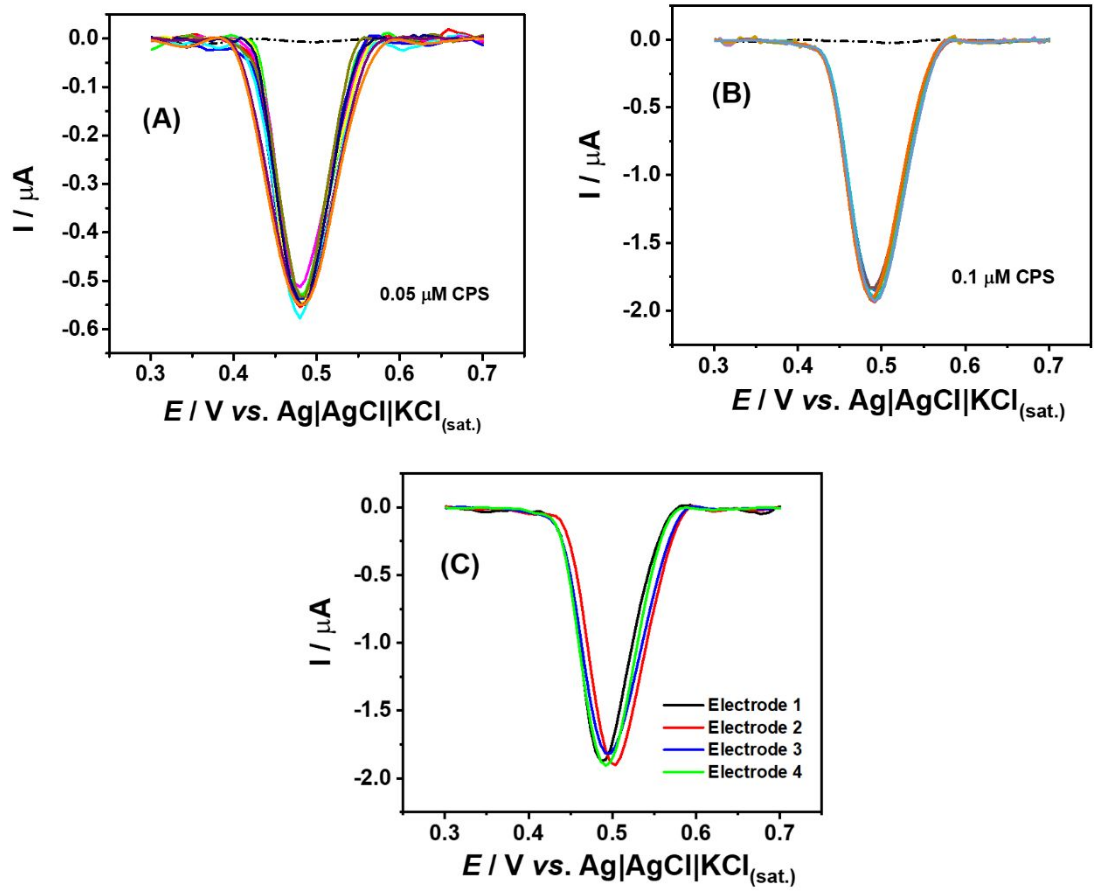

Figure S19. DPV obtained from successive measurements (n = 10) of **(A)** 0.05 µM and **(B)** 0.1 µM CPS in 0.12 M BR buffer solution (pH 2.0); **(C)** current intensities obtained for reduction process using three 3D-

CB/PLA-PT electrodes. **DPV conditions**: amplitude = 80mV, step potential = -6 mV, and modulation time = 30 ms, scan rate = 12 mV s^{-1}.

Table S8. Reproducibility study on the electrochemical behavior (peak-to-peak separation values (ΔEp) and reduction peak current (-Ipc)) using different 3D-CB/PLA-PT electrodes (n =4) under optimized conditions. The electrochemical responses were achieved for the 0.1 µM CPS in 0.12 M of BR (pH 2.0) by DPV measurements.

Electrodes	Epc /mV	-Ipc / µA
**1**	486	1.95±0.02
**2**	498	1.89±0.01
**3**	490	1.90±0.04
**4**	498	1.81±0.02

16. Surface stability study of the 3D-CB/PLA-PT electrode after measurements in the presence of CPS

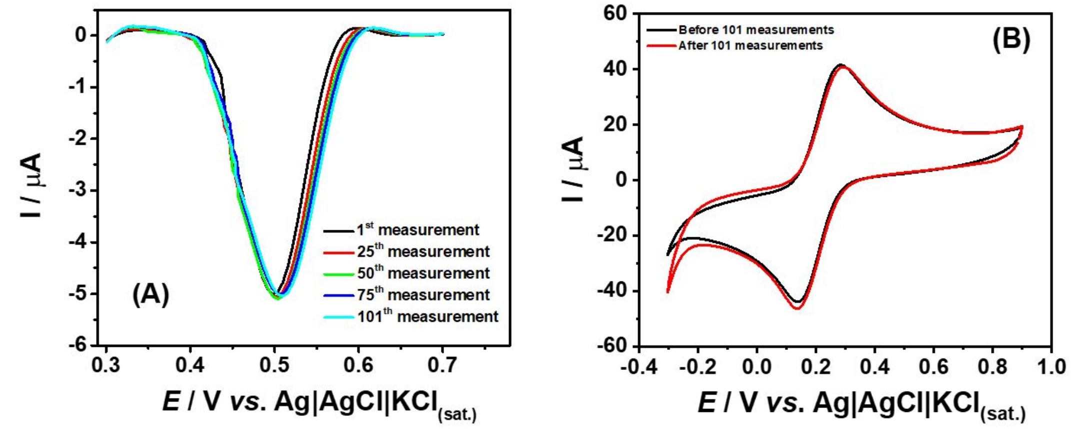

Figure S20. Stability study for the 3D-CB/PLA-PT electrode **(A)** DPV measurements of 0.5 μM CPS in 0.12 M BR buffer solution (pH 2.0) in the 1st measurement (black line), 25th measurement (red line), 50th measurement (green line), 75th measurement (dark blue line) and 101th measurement (light blue line); and **(B)** at the redox couple 2 mM $[Fe(CN)_6]^{3-/4-}$ in 0.1 M of KCl by CV, before (black line) and after (red line) conducting 101 measurements in the presence of CPS. **DPV conditions**: amplitude = 80mV, step potential = -6 mV, and modulation time = 30 ms, scan rate = 12 mV s^{-1}. **CV conditions**: scan rate = 50 mV s^{-1}; step potential = -5 mV.

17. Performance of the proposed treatment in relation to the chemical/electrochemical treatment in the presence of CPS

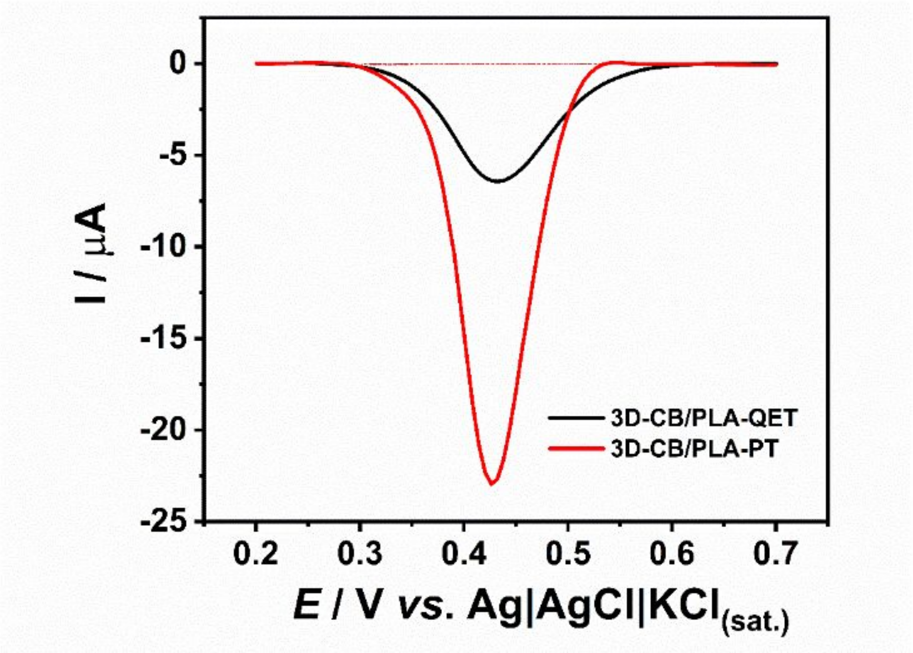

Figure S21. DPV measurements of 5.0 μM CPS in 0.12 M BR buffer solution (pH 2.0) using 3D-CB/PLA-QET (black line) and 3D-CB/PLA-PT (red line) electrodes. Electrode 3D-CB/PLA-QET was modified according to the activation protocol proposed by Richter *et al.* 2019 [12]. **DPV conditions**: amplitude = 80mV, step potential = -6 mV, and modulation time = 30 ms, scan rate = 12 mV s^{-1}.

18. Figures of merit of the electrochemical method for quantifying of CPS by DPV, and comparison of performance of the 3D-CB/PLA-PT sensor with others reported in the literature

Table S9. Analytical parameters obtained for CPS determination using 3D-CB/PLA-PT sensor and DPV technique.

Analytical Parameters	CPS
Linear range / µM	0.010 – 1.0* / 2.0 – 6.0**
R^2	0.998* / 0.986**
Intercept / µA	0.008* / -9.750**
Slope / µM µA	-10.427* / -2.729**
LOD / µM	0.003*/0.663**
LOQ / µM	0.011*/2.188**
RSD (intra-electrode, n = 10; 0.05 and 0.1 µM) /%	2.82$^{\#}$/1.91$^{\#\#}$
RSD (inter-electrode, n = 4; 0.1 µM) / %	3.00

Values for the calibration curve in the *lowest and **highest concentration range; $^{\#}$0.05 µM and $^{\#\#}$0.1 µM CPS.

Table S10. Analytical parameters (linear range and LOD) obtained using 3D-CB/PLA-PT sensor and other sensors reported for the electrochemical determination of CPS.

Electrode	Linear range (µmol L^{-1})	LOD (µmol L^{-1})	Technique	Ref.
CNTs/RuNPs/GCE	0.010 to 0.41	0.0025	SWV	29
Pencil graphite electrode	0.016 to 0.33	0.0037	SW-AdSV	30
HRP/SPE	0.75 to 25	0.30	Amperometry	31
PDDA/rGO/Pd	0.32 to 64	0.10	CV	32
IL/rGO/Nafion/GCE	0.030 to 10	0.0032	ASV	33
Unmodified SPE	0.16 to 16	0.050	DPV	34
Ag/Ag_2O/rGO/SPE	1.0 to 60	0.40	DPSV	26
Enz/MWCN/Pt electrode	20 to 100	0.61	DPV	35
10%CB SPE	0.080 to 6.0	0.028	DPV	2
3D-CB/PLA-PT	0.010 to 1.0	0.003	DPV	This work

CNTs: carbon nanotubes; **RuNPs**: ruthenium nanoparticles; **GCE**: glassy carbon electrode**; HRP**: horseradish peroxidase; **SPE**: screen-printed electrode; **PDDA**: poly dimethyl diallyl ammonium chloride; **rGO**: reduced graphene oxide; **IL**: ionic liquid; **Enz**: phenylalanineammonia-lyase enzyme; **MWCN**: multiwalled carbon nanotubes; **CB**: carbon black; **PLA**: polylactic acid; **PT**: treated with plasma; **SWV**: square wave voltammetry; **SW-AdSV**: square wave stripping voltammetric; **CV**: cyclic voltammetry; **ASV**: adsorptive linear sweep voltammetry; **DPSV**: differential pulse stripping voltammetric; **DPV**: differential pulse voltammetry.

19. Determination of CPS and recovery studies in pepper sauces samples

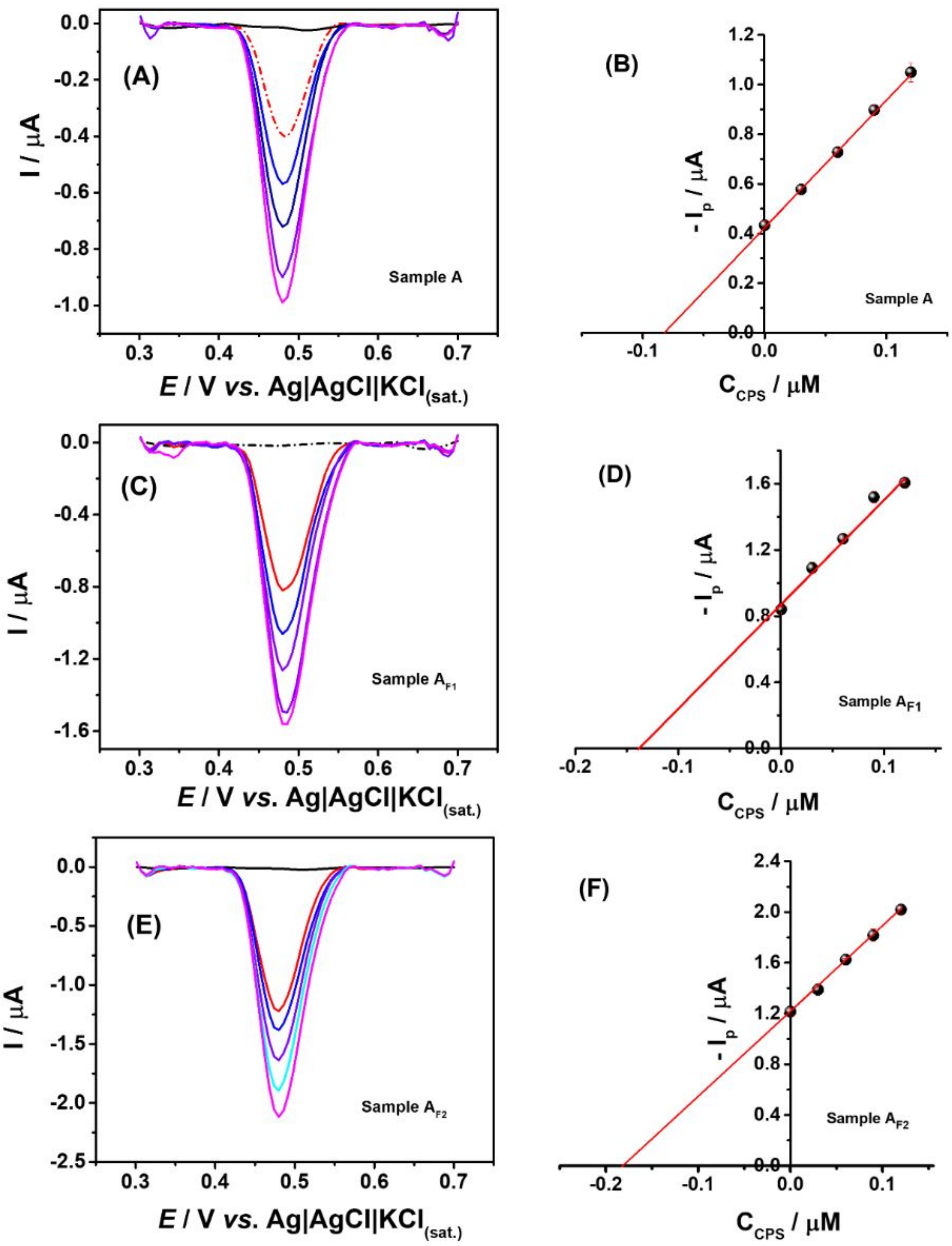

Figure S22. DPV responses were recorded for the analysis of pepper sauce sample A (red line) **(A)**; sample spiked with 0.05 µM CPS (A_{F1}, red line) **(C)** and sample spiked with 0.1 µM CPS (A_{F2}) **(E)** and successive additions of increasing concentrations of 0.03 µM CPS (Navy blue, dark blue, purple, and lilac lines). The black lines refer to the blank signal (0.12 M BR buffer solution (pH 2.0)). **(B, D, and F)** Respective calibration plots were obtained by the standard addition method. **DPV conditions**: amplitude = 80mV, step potential = -6 mV, and modulation time = 30 ms, scan rate = 12 mV s^{-1}.

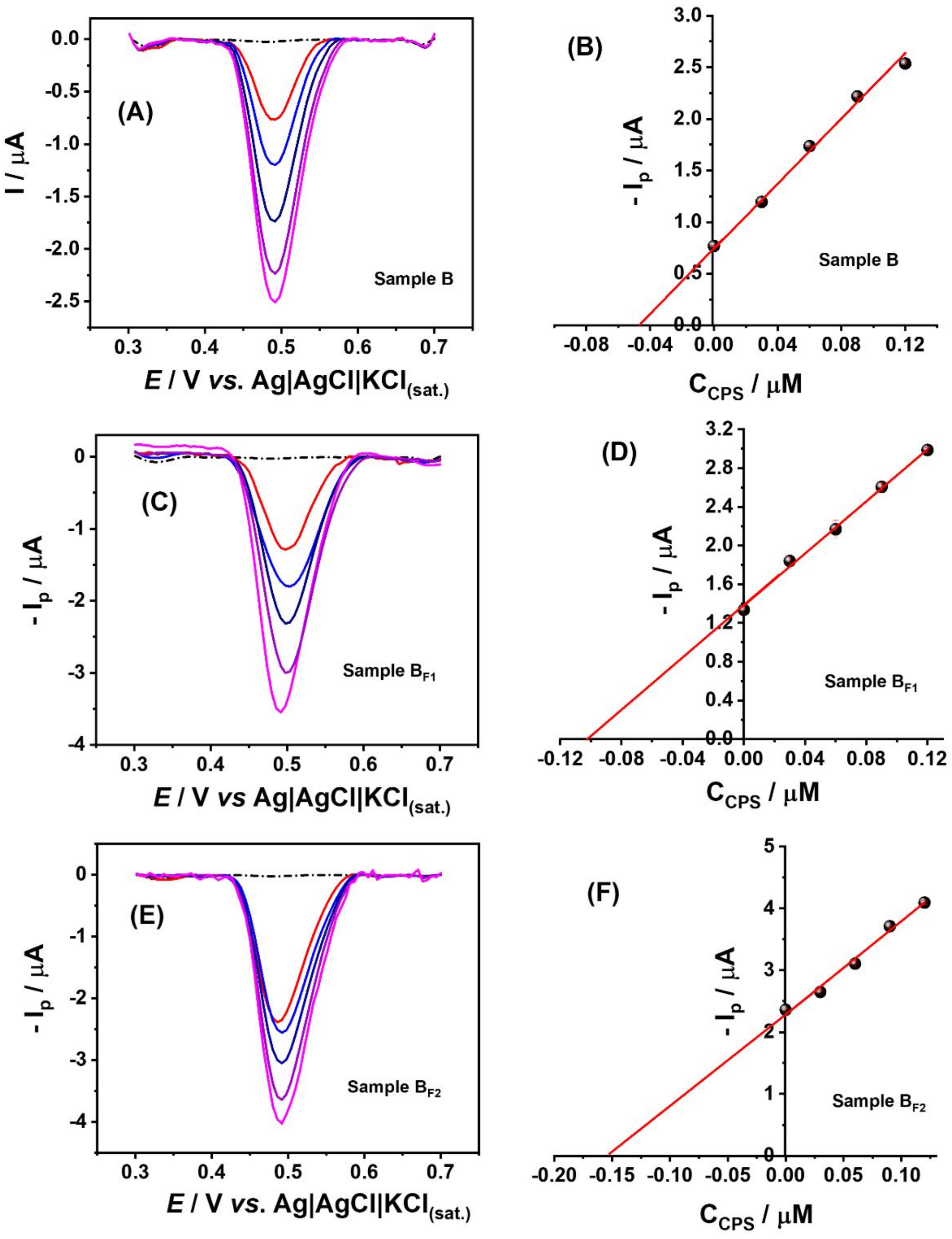

Figure S23. DPV responses recorded for the analysis of pepper sauce sample B (red line) **(A)**; sample spiked with 0.05 μM CPS (B_{F1}, red line) **(C)** and sample spiked with 0.1 μM CPS (B_{F2}) **(E)** and successive additions of increasing concentrations of 0.03 μM CPS (Navy blue, dark blue, purple, and lilac lines). The black lines refer to the blank signal (0.12 M BR buffer solution (pH 2.0)). **(B, D, and F)** Respective calibration plots were obtained by the standard addition method. DPV conditions in Table S6.

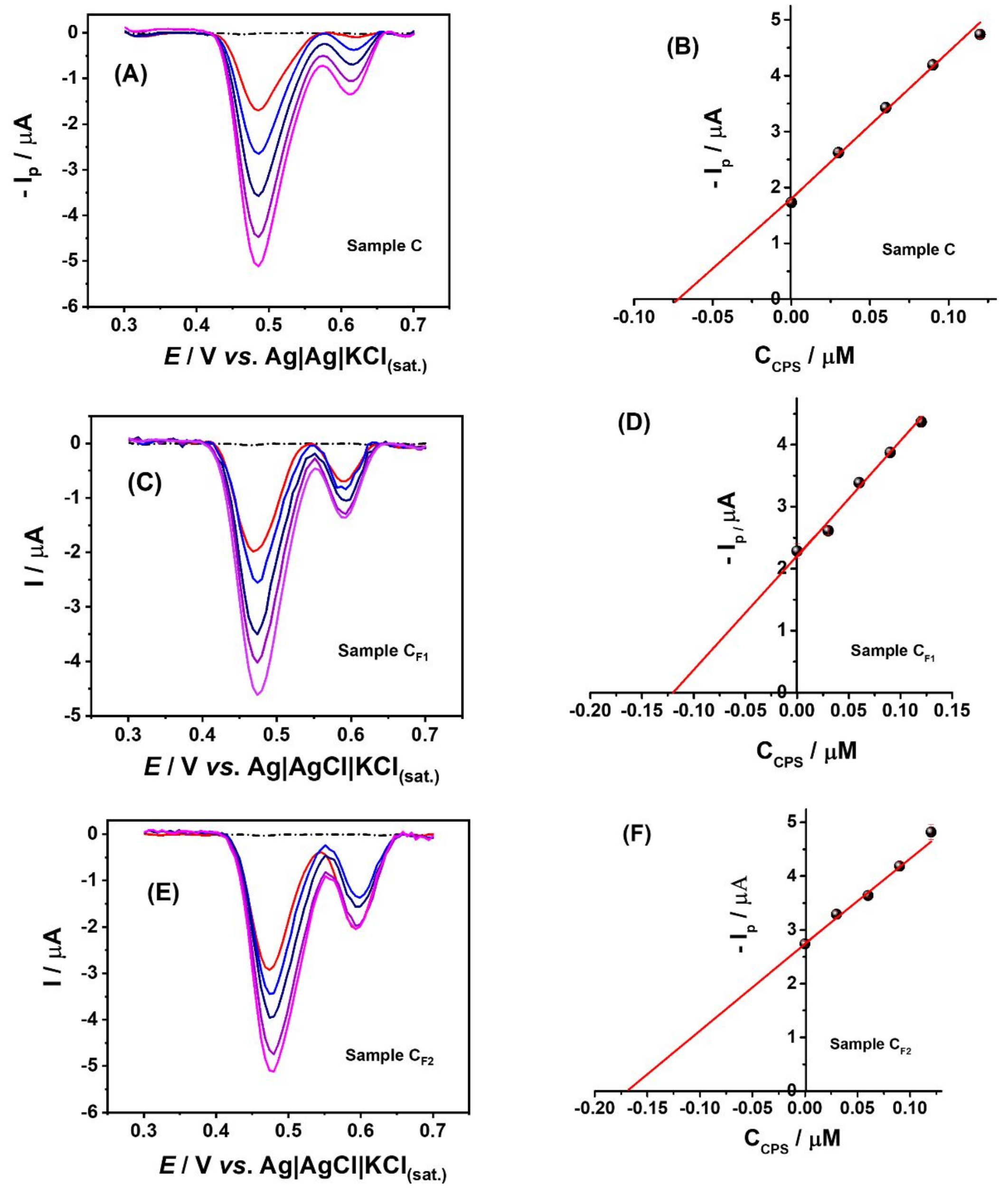

Figure S24. DPV responses recorded for the analysis of pepper sauce sample C (red line) **(A)**; sample spiked with 0.05 μM CPS (C_{F1}, red line) **(C)** and sample spiked with 0.1 μM CPS (C_{F2}) **(E)** and successive additions of increasing concentrations of 0.03 μM CPS (Navy blue, dark blue, purple, and lilac lines). The black lines refer to the blank signal (0.12 M BR buffer solution (pH 2.0)). **(B, D, and F)** Respective calibration plots were obtained by the standard addition method. DPV conditions in Table S6.

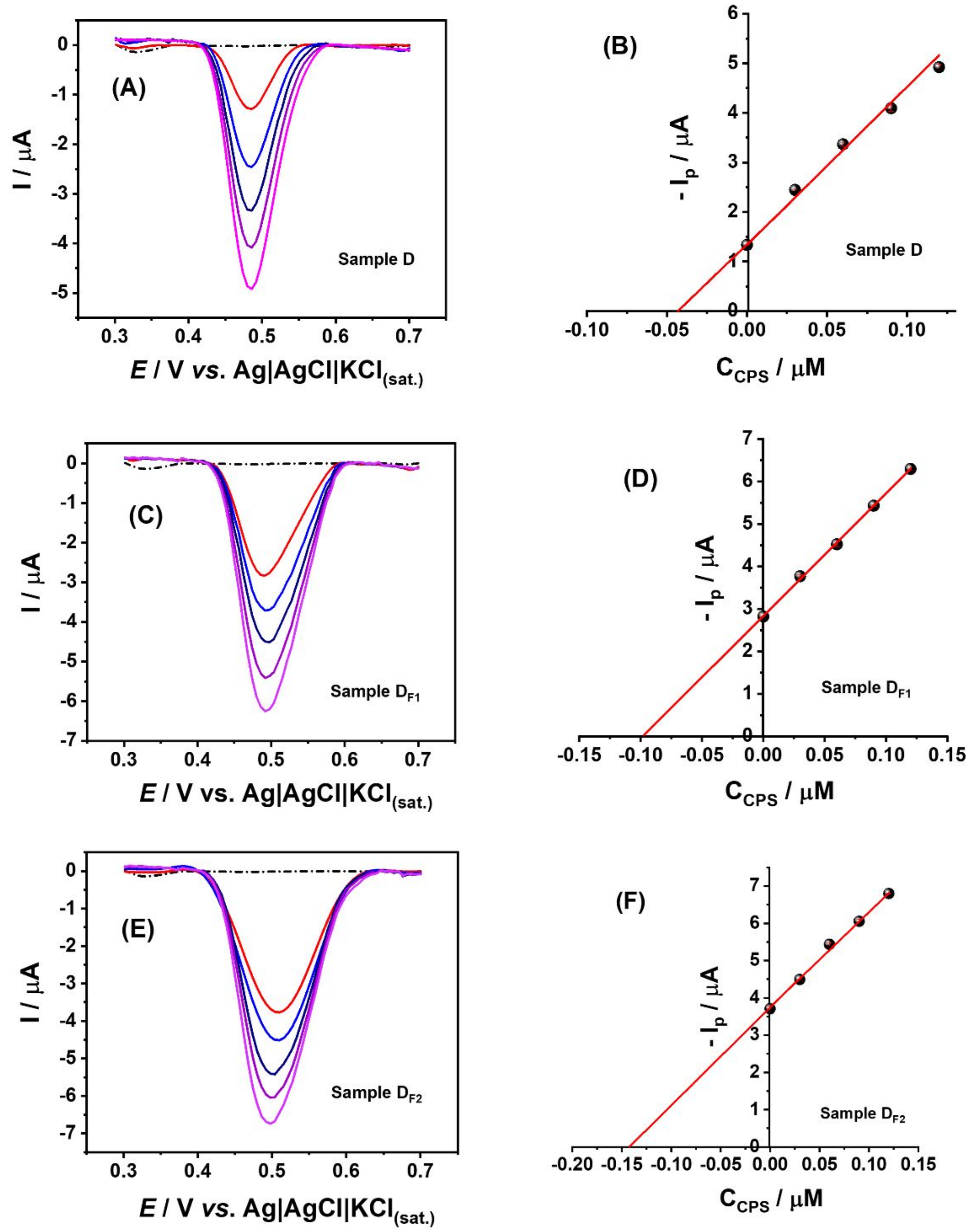

Figure S25. DPV responses recorded for the analysis of pepper sauce sample D (red line) **(A)**; sample spiked with 0.05 μM CPS (D_{F1}, red line) **(C)** and sample spiked with 0.1 μM CPS (D_{F2}) **(E)** and successive additions of increasing concentrations of 0.03 μM CPS (Navy blue, dark blue, purple, and lilac lines). The black lines refer to the blank signal (0.12 M BR buffer solution (pH 2.0)). **(B, D, and F)** Respective calibration plots were obtained by the standard addition method. DPV conditions in Table S6.

Samples A, B, C and D resulted in 0.08, 0.05, 0.07, and 0.05 µM CPS in the electrochemical cell; considering the dilution of the samples, the total capsaicinoid content in the samples is 16.6, 10.0, 14.5 and 9.9 µM, respectively.

Table S11. Results obtained in the analysis of pepper sauce samples before and after spiked with known concentrations of CPS using the 3D-CB/PLA-PT sensor.

**Samples**	**Added (µM)**	**Measured (µM)**	**Found (µM)**	**Recovery* (%)**
**A**	0.0	16.6±0.6	-	-
**A_{F1}**	10.0	26.7±0.6	10.1±0.1	101±1
**A_{F2}**	20.0	35.5±0.5	18.9±0.1	94±2
**B**	0.0	10.0±0.1	-	-
**B_{F1}**	10.0	19.8±0.9	9.8±0.4	98±4
**B_{F2}**	20.0	30.2±1.1	20.2±1.4	101±7
**C**	0.0	14.5±0.4	-	-
**C_{F1}**	10.0	24.6±0.5	10.1±0.4	101±2
**C_{F2}**	20.0	33.3±1.1	18.8±0.8	94±4
**D**	0.0	9.9±0.2	-	-
**D_{F1}**	10.0	19.8±0.3	9.9±0.2	99±3
**D_{F2}**	20.0	29.1±0.7	19.2±0.6	96±4

* Recovery (%) = [CPS] found/[CPS] added x 100.

20. References

(1) Elving, P. J.; Markowitz, J. M.; Rosenthal, I. Preparation of Buffer Systems of Constant Ionic Strength. *Anal Chem* 1956, *28* (7), 1179–1180.

(2) Deroco, P. B.; Fatibello-Filho, O.; Arduini, F.; Moscone, D. Electrochemical Determination of Capsaicin in Pepper Samples Using Sustainable Paper-Based Screen-Printed Bulk Modified with Carbon Black. *Electrochim Acta* 2020, *354*, 136628.

(3) Stefano, J. S.; Guterres e Silva, L. R.; Rocha, R. G.; Brazaca, L. C.; Richter, E. M.; Muñoz, R. A. A.; Janegitz, B. C. New Conductive Filament Ready-to-Use for 3D-Printing Electrochemical (Bio)Sensors: Towards the Detection of SARS-CoV-2. *Anal Chim Acta* 2022, *1191*, 339372.

(4) Castro, S. V. F.; Cardoso, R. M.; Santana, M. H. P.; Richter, E. M.; Munoz, R. A. A. Graphite Sheet as a Novel Material for the Collection and Electrochemical Sensing of Explosive Residues. *Talanta* 2019, *203*, 106–111.

(5) Cardoso, R. M.; Mendonça, D. M. H.; Silva, W. P.; Silva, M. N. T.; Nossol, E.; da Silva, R. A. B.; Richter, E. M.; Muñoz, R. A. A. 3D Printing for Electroanalysis: From Multiuse Electrochemical Cells to Sensors. *Anal Chim Acta* 2018, *1033*, 49–57.

(6) Siqueira, G. P.; Araújo, D. A. G.; de Faria, L. V.; Ramos, D. L. O.; Matias, T. A.; Richter, E. M.; Paixão, T. R. L. C.; Muñoz, R. A. A. A Novel 3D-Printed Graphite/Polylactic Acid Sensor for the Electrochemical Determination of 2,4,6-Trinitrotoluene Residues in Environmental Waters. *Chemosphere* 2023, *340*, 139796.

(7) Bin Hamzah, H. H.; Keattch, O.; Covill, D.; Patel, B. A. The Effects of Printing Orientation on the Electrochemical Behaviour of 3D Printed Acrylonitrile Butadiene Styrene (ABS)/Carbon Black Electrodes. *Sci Rep* 2018, *8* (1), 9135.

(8) Areir, M.; Xu, Y.; Zhang, R.; Harrison, D.; Fyson, J.; Pei, E. A Study of 3D Printed Active Carbon Electrode for the Manufacture of Electric Double-Layer Capacitors. *J Manuf Process* 2017, *25*, 351–356.

(9) Zhu, B.; Yu, L.; Beikzadeh, S.; Zhang, S.; Zhang, P.; Wang, L.; Travas-Sejdic, J. Disposable and Portable Gold Nanoparticles Modified - Laser-Scribed Graphene Sensing Strips for Electrochemical, Non-Enzymatic Detection of Glucose. *Electrochim Acta* 2021, *378*, 138132.

(10) Chang, Z.; Zhu, B.; Liu, J.; Zhu, X.; Xu, M.; Travas-Sejdic, J. Electrochemical Aptasensor for 17β-Estradiol Using Disposable Laser Scribed Graphene Electrodes. *Biosens Bioelectron* 2021, *185*, 113247.

(11) Wang, Y.; Limon-Petersen, J. G.; Compton, R. G. Measurement of the Diffusion Coefficients of [Ru(NH3)6]3+ and [Ru(NH3)6]2+ in Aqueous Solution Using Microelectrode Double Potential Step Chronoamperometry. *J Electroanal Chem* 2011, *652* (1–2), 13–17.

(12) Richter, E. M.; Rocha, D. P.; Cardoso, R. M.; Keefe, E. M.; Foster, C. W.; Munoz, R. A. A.; Banks, C. E. Complete Additively Manufactured (3D-Printed) Electrochemical Sensing Platform. *Anal Chem* 2019, *91* (20), 12844–12851.

(13) Mocak; A., M. A.; Mitchell; Scollaryb, G. A Statistical Overview of Standard (IUPAC and ACS) and New Procedures for Determining the Limits of Detection and Quantification: Application to Voltammetric and Stripping Techniques (Technical Report). *Pure Appl Chem* 1997, *69* (2), 297–328.

(14) Shin, J.; Seo, K.; Park, H.; Park, D. Performance Improvement of Acid Pretreated 3D-printing Composite for the Heavy Metal Ions Analysis. *Electroanalysis* 2021, *33* (7), 1707–1714.

(15) Redondo, E.; Muñoz, J.; Pumera, M. Green Activation Using Reducing Agents of Carbon-Based 3D Printed Electrodes: Turning Good Electrodes to Great. *Carbon N Y* 2021, *175*, 413–419.

(16) Pereira, J. F. S.; Rocha, R. G.; Castro, S. V. F.; João, A. F.; Borges, P. H. S.; Rocha, D. P.; de Siervo, A.; Richter, E. M.; Nossol, E.; Gelamo, R. V.; Muñoz, R. A. A. Reactive Oxygen Plasma Treatment of 3D-Printed Carbon Electrodes towards High-Performance Electrochemical Sensors. *Sens Actuators B Chem* 2021, *347*, 130651.

(17) Novotný, F.; Urbanová, V.; Plutnar, J.; Pumera, M. Preserving Fine Structure Details and Dramatically Enhancing Electron Transfer Rates in Graphene 3D-Printed Electrodes via Thermal Annealing: Toward Nitroaromatic Explosives Sensing. *ACS Appl Mater Interfaces* 2019, *11* (38), 35371–35375.

(18) Browne, M. P.; Novotný, F.; Sofer, Z.; Pumera, M. 3D Printed Graphene Electrodes' Electrochemical Activation. *ACS Appl Mater Interfaces* 2018, *10* (46), 40294–40301.

(19) Manzanares-Palenzuela, C. L.; Hermanova, S.; Sofer, Z.; Pumera, M. Proteinase-Sculptured 3D-Printed Graphene/Polylactic Acid Electrodes as Potential Biosensing Platforms: Towards Enzymatic Modeling of 3D-Printed Structures. *Nanoscale* 2019, *11* (25), 12124–12131.

(20) dos Santos, P. L.; Katic, V.; Loureiro, H. C.; dos Santos, M. F.; dos Santos, D. P.; Formiga, A. L. B.; Bonacin, J. A. Enhanced Performance of 3D Printed Graphene Electrodes after Electrochemical Pre-Treatment: Role of Exposed Graphene Sheets. *Sens Actuators B Chem* 2019, *281*, 837–848.

(21) Rocha, D. P.; Ataide, V. N.; de Siervo, A.; Gonçalves, J. M.; Muñoz, R. A. A.; Paixão, T. R. L. C.; Angnes, L. Reagentless and Sub-Minute Laser-Scribing Treatment to Produce Enhanced Disposable Electrochemical Sensors via Additive Manufacture. *Chem Eng J* 2021, *425*, 130594.

(22) Glowacki, M. J.; Cieslik, M.; Sawczak, M.; Koterwa, A.; Kaczmarzyk, I.; Jendrzejewski, R.; Szynkiewicz, L.; Ossowski, T.; Bogdanowicz, R.; Niedzialkowski, P.; Ryl, J. Helium-Assisted, Solvent-Free Electro-Activation of 3D Printed Conductive Carbon-Polylactide Electrodes by Pulsed Laser Ablation. *Appl Surf Sci* 2021, *556*, 149788.

(23) Rocha, D. P.; Squissato, A. L.; da Silva, S. M.; Richter, E. M.; Munoz, R. A. A. Improved Electrochemical Detection of Metals in Biological Samples Using 3D-Printed Electrode: Chemical/Electrochemical Treatment Exposes Carbon-Black Conductive Sites. *Electrochim Acta* 2020, *335*, 1–11.

(24) Veloso, W. B.; Ataide, V. N.; Rocha, D. P.; Nogueira, H. P.; de Siervo, A.; Angnes, L.; Muñoz, R. A. A.; Paixão, T. R. L. C. 3D-Printed Sensor Decorated with Nanomaterials by CO2 Laser Ablation and Electrochemical Treatment for Non-Enzymatic Tyrosine Detection. *Microchim Acta* 2023, *190* (2), 63.

(25) Kachoosangi, R. T.; Wildgoose, G. G.; Compton, R. G. Carbon Nanotube-Based Electrochemical Sensors for Quantifying the 'Heat' of Chilli Peppers: The Adsorptive Stripping Voltammetric Determination of Capsaicin. *Analyst* 2008, *133* (7), 888.

(26) Wang, Y.; Huang, B.; Dai, W.; Ye, J.; Xu, B. Sensitive Determination of Capsaicin on Ag/Ag2O Nanoparticles/Reduced Graphene Oxide Modified Screen-Printed Electrode. *J Electroanal Chem* 2016, *776*, 93–100.

(27) Yardım, Y. Sensitive Detection of Capsaicin by Adsorptive Stripping Voltammetry at a Boron-Doped Diamond Electrode in the Presence of Sodium Dodecylsulfate. *Electroanalysis* 2011, *23* (10), 2491–2497.

(28) Junior Gosser, D. K. Cyclic Voltammetry; Simulation and Analysis of Reaction Mechanisms. *Wiley-VCH* 1993, *24* (7), 1237–1238.

(29) Baytak, A. K.; Aslanoglu, M. Sensitive Determination of Capsaicin in Pepper Samples Using a Voltammetric Platform Based on Carbon Nanotubes and Ruthenium Nanoparticles. *Food Chem* 2017, *228*, 152–157.

(30) Yardım, Y.; Şentürk, Z. Electrochemical Evaluation and Adsorptive Stripping Voltammetric Determination of Capsaicin or Dihydrocapsaicin on a Disposable Pencil Graphite Electrode. *Talanta* 2013, *112*, 11–19.

(31) Mohammad, R.; Ahmad, M.; Heng, L. Y. Amperometric Capsaicin Biosensor Based on Covalent Immobilization of Horseradish Peroxidase (HRP) on Acrylic Microspheres for Chilli Hotness Determination. *Sens Actuators B Chem* 2017, *241*, 174–181.

(32) Zhong, F.; Liu, Z.; Han, Y.; Guo, Y. Electrochemical Sensor for Sensitive Determination of Capsaicin Using Pd Decorated Reduced Graphene Oxide. *Electroanalysis* 2019, *31* (6), 1182–1188.

(33) Kim, D.-H.; Nam, S.; Kim, J.; Lee, W.-Y. Electrochemical Determination of Capsaicin by Ionic Liquid Composite-Modified Electrode. *J Electrochem. Sci Technol* 2019, *10* (2), 177–184.

(34) Lyu, W.; Zhang, X.; Zhang, Z.; Chen, X.; Zhou, Y.; Chen, H.; Wang, H.; Ding, M. A Simple and Sensitive Electrochemical Method for the Determination of Capsaicinoids in Chilli Peppers. *Sens Actuators B Chem* 2019, *288*, 65–70.

(35) Sabela, M. I.; Mpanza, T.; Kanchi, S.; Sharma, D.; Bisetty, K. Electrochemical Sensing Platform Amplified with a Nanobiocomposite of L-Phenylalanine Ammonia-Lyase Enzyme for the Detection of Capsaicin. *Biosens Bioelectron* 2016, *83*, 45–53.
